# Genotypic variation in maize leaf senescence after ear removal is caused by excess accumulation of photosynthates

**DOI:** 10.1093/plphys/kiag190

**Published:** 2026-04-08

**Authors:** Jie Yuan, Fengying Duan, Kaijian Fan, Yanfang Heng, Xuxiang Wang, Yupeng Zhou, Qiang He, Shaobo Wei, Xuefang Sun, Liang Li, Xia Li, Wenbin Zhou

**Affiliations:** State Key Laboratory of Crop Gene Resources and Breeding, Institute of Crop Sciences, Chinese Academy of Agricultural Sciences, Beijing 100081, China; College of Agronomy, Qingdao Agricultural University, Qingdao 266109, China; State Key Laboratory of Crop Gene Resources and Breeding, Institute of Crop Sciences, Chinese Academy of Agricultural Sciences, Beijing 100081, China; State Key Laboratory of Crop Gene Resources and Breeding, Institute of Crop Sciences, Chinese Academy of Agricultural Sciences, Beijing 100081, China; State Key Laboratory of Crop Gene Resources and Breeding, Institute of Crop Sciences, Chinese Academy of Agricultural Sciences, Beijing 100081, China; State Key Laboratory of Crop Gene Resources and Breeding, Institute of Crop Sciences, Chinese Academy of Agricultural Sciences, Beijing 100081, China; State Key Laboratory of Crop Gene Resources and Breeding, Institute of Crop Sciences, Chinese Academy of Agricultural Sciences, Beijing 100081, China; State Key Laboratory of Crop Gene Resources and Breeding, Institute of Crop Sciences, Chinese Academy of Agricultural Sciences, Beijing 100081, China; State Key Laboratory of Crop Gene Resources and Breeding, Institute of Crop Sciences, Chinese Academy of Agricultural Sciences, Beijing 100081, China; College of Agronomy, Qingdao Agricultural University, Qingdao 266109, China; State Key Laboratory of Crop Gene Resources and Breeding, Institute of Crop Sciences, Chinese Academy of Agricultural Sciences, Beijing 100081, China; State Key Laboratory of Crop Gene Resources and Breeding, Institute of Crop Sciences, Chinese Academy of Agricultural Sciences, Beijing 100081, China; State Key Laboratory of Crop Gene Resources and Breeding, Institute of Crop Sciences, Chinese Academy of Agricultural Sciences, Beijing 100081, China

## Abstract

Leaf senescence is a key developmental process influencing photosynthesis, carbon allocation, and crop productivity, all tightly regulated by source–sink balance. In maize (*Zea mays*), abscisic acid signaling and nonstructural carbohydrate partitioning have been proposed as major determinants of senescence timing under sink limitation. Through multiyear, multigenotype analyses, we refine the current understanding by identifying starch-induced chloroplast disruption as the primary trigger of sink-removal-induced senescence, whereas abscisic acid acts mainly as a secondary, modulatory signal. We also reveal a respiration-linked metabolic compensation mechanism that stabilizes leaf function in genotypes with delayed senescence. In early-senescing genotypes, ear removal induced significant chlorophyll loss, anthocyanin accumulation, and severe sugar/starch overaccumulation that disrupted chloroplast ultrastructure, coinciding with reduced leaf protein contents and impaired photosynthesis. By contrast, genotypes with delayed senescence maintained pigment stability, displaying enhanced dark respiration, balanced carbohydrate metabolism, and chloroplast integrity. Shading experiments further confirmed that starch overaccumulation, rather than abscisic acid elevation, is the dominant driver of sink-removal-induced senescence. Metabolomic analyses showed that early-senescing genotypes accumulate soluble sugars and phosphoenolpyruvate-pathway intermediates, whereas genotypes with delayed senescence exhibited reduced tricarboxylic acid cycle activity and elevated aspartate-family amino acids. Transcriptomics revealed downregulation of sucrose transporters (eg *ZmSUT7*) and starch degradation genes (eg *ZmBmy3*) alongside upregulation of anthocyanin and carbohydrate metabolism genes in early-senescing genotypes. Coexpression network analysis further identified hub genes associated with carbohydrate and amino acid metabolism, linking transcriptional regulation to genotype-specific metabolic adjustments. Together, these findings refine current source–sink regulatory models and offer potential physiological and metabolic targets for improving source–sink coordination and enhancing yield resilience in maize.

## Teaser

Comparisons of maize varieties reveal that excess starch buildup triggers leaf senescence after sink removal and highlight metabolic features that refine understanding of source–sink regulation.

## Introduction

Leaf senescence is a finely regulated degenerative process that plays a critical role in nutrient recycling and remobilization to developing organs, thereby significantly influencing crop growth and yield formation (Guo et al. 2021). Senescence is orchestrated by an intricate network of internal signals, including hormones such as cytokinin, abscisic acid (ABA), ethylene, salicylic acid, gibberellic acid, and auxins, as well as carbohydrate signaling, which can act in a context-dependent manner to either promote or delay senescence (Ahmad and Guo 2019; Kim 2019; Woo et al. 2019; Asad et al. 2024).

A central determinant of senescence onset is the source–sink relationship, which governs the allocation of photoassimilates from photosynthetic tissues (sources) to developing or storage organs (sinks) (Mason and Maskell 1928; Rosado-Souza et al. 2023; Smith et al. 2018). The efficiency of this process depends on phloem transport (flow) and is influenced by the strength of the source (eg photosynthetic capacity, nutrient uptake) and sink demand (eg metabolic capacity, storage activities) (Arp 1991; De Schepper et al. 2013). Enhancing both aspects synergistically boosts yield, as demonstrated by coordinated “push-pull” strategies that doubled tuber yield and starch accumulation in potato (Jonik et al. 2012).

When source–sink balance is perturbed, senescence can be triggered prematurely. A weakened source may cause carbohydrate depletion, whereas reduced sink demand may lead to carbohydrate overaccumulation; in either case, homeostasis is disrupted and leaf senescence is accelerated (Muller et al. 2011; Abeledo et al. 2020). To probe these mechanisms, experimental manipulations such as ear or floral removal, fruit thinning, or altered temperature treatments have been widely applied. These studies revealed species-specific and genotype-dependent senescence responses (Lv et al. 2020). For instance, pod removal in soybean delayed leaf senescence by maintaining chlorophyll and sugar levels (Zhang et al. 2016; Yun et al. 2020), with similar effects in sunflower, cowpeas, and wheat (Biswas and Mandal 1986; Ho et al. 1987; Khanna-Chopra and Reddy 1988). In contrast, sink removal in maize and barley often accelerates leaf senescence (Christensen et al. 1981; Crafts-Brandner et al. 1984).

Notably, senescence responses in maize are strongly genotype-dependent. For example, the inbred line B73 displays early senescence after ear removal, whereas Mo17 retains greenness (Ceppi et al. 1987; Sekhon et al. 2012). A broader screen identified only 3 stay-green inbred lines among 31 tested under nonpollinated conditions (Kumar et al. 2019), highlighting substantial natural variation in this trait. Recent multiomics studies in maize have advanced our understanding of source–sink regulated senescence, linking carbohydrate accumulation, trehalose-6-phosphate (T6P) signaling, and extensive transcriptomic reprogramming to senescence progression (Sekhon et al. 2012; Wu et al. 2018). These changes are accompanied by transcriptomic and metabolomic reprogramming in pathways related to proteolysis, oxidative stress, ABA signaling, and protein homeostasis (Kumar et al. 2023). Nevertheless, the genetic and mechanistic basis underlying genotype-specific senescence responses to sink manipulation in maize remains poorly understood.

In this study, we investigated 6 maize genotypes with contrasting senescence behaviors under ear-removal conditions, exhibiting early- or delay-senescing phenotypes. By integrating physiological, anatomical, metabolomic, and transcriptomic analyses, we dissected the mechanistic basis of these divergent responses and identified key regulatory pathways and candidate genes. Our findings offer insights into the genetic and metabolic regulation of source–sink regulated senescence and provide a foundation for improving source–sink coordination in maize breeding.

## Materials and methods

### Plant materials and experimental design

Six maize (*Zea mays* L.) genotypes were used in this study, including Xianyu335 (XY335), PH6WC, PH4CV, Zhengdan958 (ZD958), Zheng58 (Z58), and Chang7-2 (C7-2). Both XY335 and ZD958 are major maize hybrid varieties widely cultivated in China, with contrasting responses to ear removal. XY335 (including its parental inbred lines PH6WC and PH4CV) displays early leaf senescence following ear removal, while ZD958 (composed of parental lines Z58 and C7-2) exhibits a delay-senescing phenotype under the same condition.

Field trials were conducted during the summer of 2022–2024 in Beijing (N 40°10′47″, E 116°14′49″), where the maize life cycle spans ∼120 days. ZD958 reaches silking in ∼55 days postemergence, while XY335 silks 3–5 days later. Prior to silking, ears were bagged to prevent cross-pollination. Uniformly growing plants were tagged and pollinated on the same day after silking. Two treatments were applied: Ear+ (Control)—normal pollination without intervention, and Ear- (Ear Removal)—female ears were removed immediately after pollination. To investigate the impact of leaf shading after ear removal, approximately one-fourth of the ear leaf area was covered with tinfoil to impose shading.

The experiment followed a randomized block design with 3 biological replicates. Each plot measured 4.2 m × 2 m, with a planting density of 60 cm (row spacing) × 20 cm (plant spacing). Standard agronomic practices were used for irrigation, fertilization, and pest/disease/weed control.

In most experiments, each biological replicate was generated by pooling samples from 3 plants. For the shading experiment, protein content determination, gas exchange measurements, histological analysis, TEM, and biomass accumulation and distribution assays, each biological replicate corresponded to an individual plant.

### Pigment measurements

For both Ear+ and Ear- treatments, leaves at the ear position were collected at 1, 9, 19, and 25 DAT and immediately frozen in liquid nitrogen. Chlorophyll and carotenoid were extracted with ground leaf samples in 100% acetone under dim light. Absorbance values at 470, 644.8, and 661.6 nm were recorded using a spectrophotometer (Ultrospec 7000, Biochrom). Chlorophyll and carotenoid concentrations were determined as described by Lichtenthaler (1987).

Approximately 30 mg of fresh leaf tissue was ground and extracted in 450 μL acidified methanol (99:1 methanol:HCl). Anthocyanins were extracted by incubating the samples at 4 °C in darkness for 10 h. After adding 300 μL deionized water and 750 μL chloroform, the mixture was centrifuged, and the absorbance of the supernatant was measured at 530 and 657 nm using a spectrophotometer (Ultrospec 7000, Biochrom, USA) as described previously (Nakata et al. 2013).

### Sugar and starch content measurements

Carbohydrate content was determined following Li et al. (2020). Leaves from normal pollination and ear removal treatments were sampled at 1, 9, 19, and 25 DAT, as well as from shaded and unshaded regions after 30 days of shading, then rapidly frozen in liquid nitrogen. Approximately 50 mg of leaf tissue was extracted with 1 mL 80% (v/v) ethanol at 80 °C for 30 min, centrifuged, and repeated twice. Supernatants were combined, vacuum-dried, dissolved in deionized water, and used to assay sucrose, fructose, and glucose using Sucrose, D-fructose, and Glucose Assay Kits (Megazyme). Residual pellets were used for starch quantification with the Total Starch Assay Kit (Megazyme).

### Protein content determination

Maize leaf samples were collected at 25 days after Ear- treatment. Samples from Ear+ group were collected at the same time. For protein extraction, 0.1 g of fresh leaf tissue was homogenized in 400 μL of protein extraction buffer. The homogenate was incubated with shaking at 40 rpm for 30 min at room temperature, followed by centrifugation at 12000 rpm for 20 min at 4 °C. The supernatant was collected and protein concentration was determined using Quick Start Bradford Reagent (Bio-Rad, USA). All extraction procedures were performed on ice to maintain protein stability.

### Gas exchange measurements

Gas exchange was measured with ear leaves on the 25th day after normal pollination and ear removal treatment, using the LI-6400XT system (LI−COR Inc., Lincoln, NE, USA), from 9:00–11:30 AM under a photosynthetic photon flux density (PPFD) of 1500 µmol photons m^−2^ s^−1^. Dark respiration (Rd) rates were assessed between 21:00 and 24:00 at 0 PPFD.

### Histological analysis

Leaves collected at 25 DAT were cut into 15 × 5 mm sections and fixed in precooled FAA (formaldehyde: acetic acid: ethanol = 5:5:90 [v/v]). Fixed tissues were paraffin-embedded and sectioned (8 μm) using an ultrathin microtome (Ultra-cut R, Leica). Sections were then stained with Senna solid green and visualized under a light microscope (DM5500B, Leica). Leaf thickness and vascular bundle area were quantified using ImageJ software.

### Transmission electron microscopy

Leaf segments (1 × 1 mm) were collected at 25 DAT and 30 days after shading. Samples were immediately fixed in 2% glutaraldehyde (pH 7.2), then immersed in 1% osmium tetroxide at 4 °C for 20 h. Following dehydration in ethanol and acetone series, samples were then subjected to vacuum drying in propylene oxide twice. After embedding and ultrathin sectioning, the samples were viewed with a transmission electron microscope (Hitachi H-7650). Quantitative analysis of chloroplasts, osmiophilic granules, and starch granules was conducted using ImageJ software.

### Abscisic acid quantification

ABA content of ear leaves was measured at 1, 9, and 25 DAT according to the published protocol (Soualiou et al. 2023).

### Biomass accumulation and distribution

Six representative plants showing red leaf coloration after Ear- treatment were selected. Samples were initially dried at 110 °C for 15 min to deactivate enzymes, followed by oven-drying at 80 °C until achieving constant weight. Dry weight of root (RDW), stem (SDW), and leaf (LDW) were measured separately. Total biomass = RDW + SDW + LDW. Percentage of DW increase was calculated as: (total biomass in Ear- treatment – total biomass in Ear+ group)/total biomass in Ear+ group.

### Metabolite profiling

Maize leaves sampled at 19 and 25 DAT were used for metabolite profiling. Leaf samples (20 mg) were lyophilized, extracted with a methanol/acetonitrile/water (2:2:1) solution containing deuterated internal standards, homogenized, sonicated, and centrifuged to obtain the supernatant for analysis, with QC samples prepared by pooling equal aliquots of the extracts. Metabolites were identified by matching MS and MS/MS data (<30 ppm) against databases including HMDB, MassBank, LipidMaps, mzCloud, and KEGG. Robust LOESS signal correction (QC-RLSC) was applied for data normalization to correct for systematic bias. The statistical significance of metabolites was assessed using a combination of criteria. *P* values were derived from appropriate hypothesis tests, while variable importance in projection (VIP) scores were obtained from orthogonal partial least squares-discriminant analysis. Additionally, fold change was calculated to quantify the magnitude of differences between groups. These metrics collectively evaluated the contribution and explanatory power of each metabolite in sample classification and discrimination, thereby facilitating the identification of potential biomarker metabolites. A metabolite was considered statistically significant when *P* < 0.05 and VIP > 1. Differential metabolites were further analyzed for pathway enrichment using MetaboAnalyst. The metabolites and their corresponding pathways were visualized using the KEGG Mapper tool.

### Transcriptome analysis

Maize leaf samples were collected at 9, 19, and 25 DAT. Total RNA was extracted using TRIzol ® reagent (Invitrogen), and RNA-seq libraries were constructed by using the TruSeq Stranded mRNA LT Sample Prep Kit (Illumina, USA). Sequencing was performed on an Illumina HiSeq platform. Clean reads were aligned to the maize B73 reference genome (AGPv4) using HISAT2 (v2.1.0) (Kim et al. 2015), and transcript abundance was quantified with StringTie (v1.3.3b) (Pertea et al. 2015). DEGs were identified with the DESeq2 R package (Love et al. 2014), applying thresholds (*adj. P*-value < 0.05 and |log_2_ fold change| ≥ 1) to identify statistically significantly different expression in Ear- treated maize versus normally pollinated (Ear+) plants. KEGG enrichment was conducted using clusterProfiler (Wu et al. 2021). WGCNA was performed in R using the WGCNA package (Langfelder and Horvath 2008), and the regulatory networks were visualized with Cytoscape. Raw sequence data generated in this study have been deposited in the NCBI BioProject database under accession number PRJNA1340406.

### Reverse transcription quantitative PCR

Total RNA was extracted from leaves at 25 DAT using the TRIzol reagent (Invitrogen). For cDNA synthesis, 1 μg total RNA was used as template for reverse transcription following a 2-step procedure using *Evo M*-*MLV* RT Mix Kit with gDNA Clean for reverse transcription quantitative PCR (RT-qPCR) (Accurate Biology, China). RT-qPCR was performed with SYBR Green *Pro Taq* HS premix on an ABI QuantStudio 6 Flex system (Applied Biosystems, USA). Primer sequences are listed in Table S9.

## Results

### Genotypic variation in maize leaf senescence following ear removal

**Figure 1:**
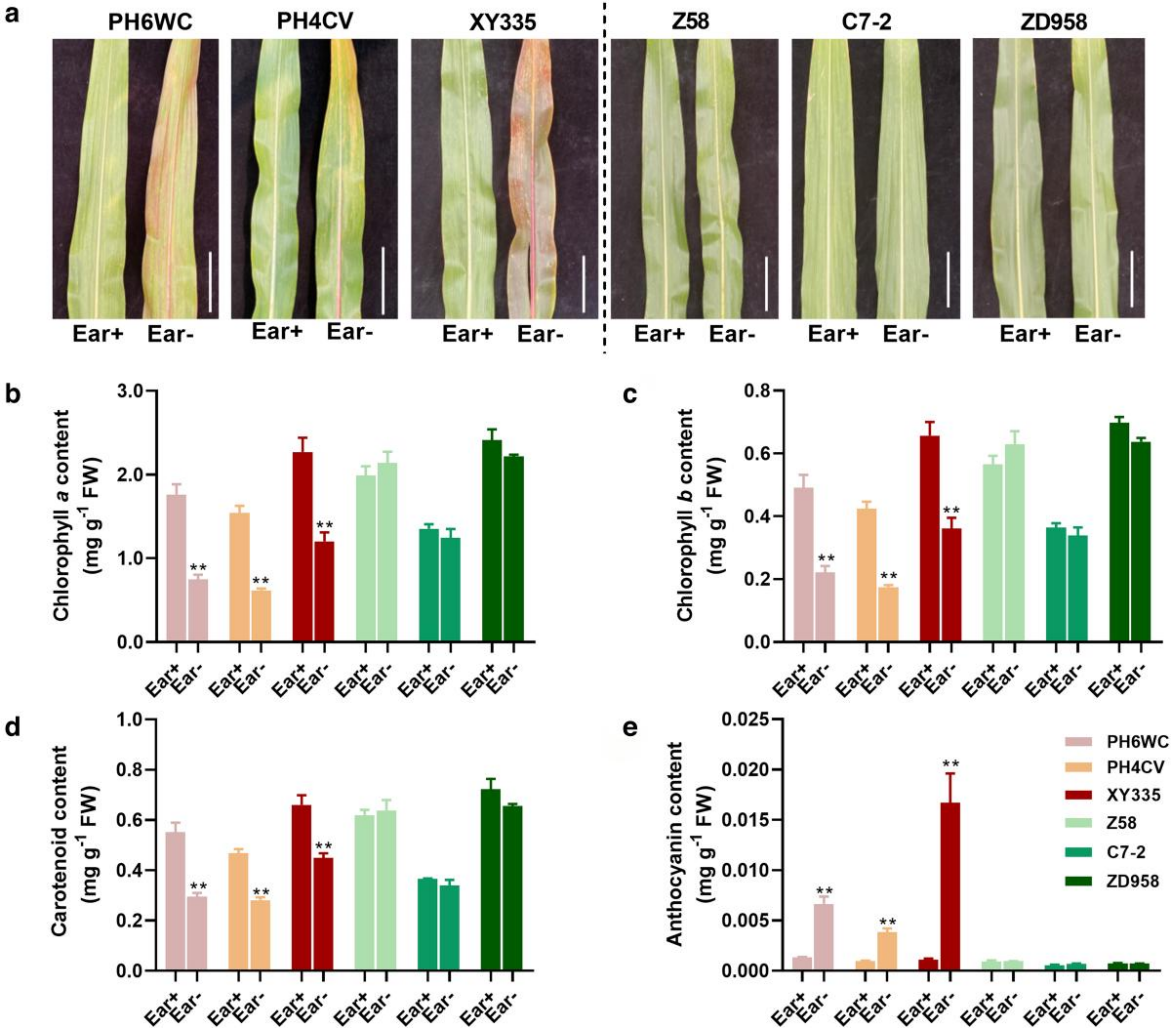
Ear leaf phenotype and pigment content in 6 maize genotypes at 25 DAT under normal pollination (Ear+) and ear removal (Ear-) treatments. (a) Leaf phenotypes of PH6WC, PH4CV, XY335, Z58, C7-2, and ZD958 at 25 DAT under Ear+ and Ear- treatments. (b-e) Contents of chlorophyll *a* (b), chlorophyll *b* (c), carotenoids (d), and anthocyanins (e) in ear leaves under Ear+ and Ear- treatments. Data are presented as means ± SEM (*n* ≥ 3). ** indicates a statistically significant difference at *P* ≤ 0.01 compared with Ear+ (Student's *t*-test). Scale bar = 10 cm. DAT, days after treatment.

To investigate genotypic variation in leaf senescence triggered by ear removal, 6 genetically distinct maize genotypes were selected for phenotypic and physiological characterization. Notably, the hybrid Xianyu 335 (XY335) and its parental lines, PH6WC and PH4CV, exhibited clear signs of premature senescence following ear removal. By 19 days after treatment (DAT), their ear leaves had developed visible red pigmentation along the main veins, which intensified with time. By 25 DAT, most leaves had turned red and displayed extensive senescence. In contrast, the hybrid Zhengdan 958 (ZD958) and its parental lines, Zheng58 (Z58) and Chang7-2 (C7-2), showed no apparent phenotypic alterations, maintaining green leaf coloration throughout the experimental period and exhibiting a pronounced delay-senescing phenotype (Fig. 1a).

To quantitatively assess these phenotypic changes, pigment contents in ear leaves were measured. In XY335, PH6WC, and PH4CV, significant reductions in chlorophyll *a*, chlorophyll *b*, and carotenoid contents were observed under the ear removal (Ear-) treatment compared to the normally pollinated control (Ear+) at 25 DAT. Concurrently, anthocyanin content was markedly elevated under Ear- treatment in these genotypes (Fig. 1b-e). By contrast, pigment levels remained unaffected in Z58, C7-2, and ZD958. Time-course analysis of pigment ratios (Ear-/Ear+) further highlighted the divergent responses among the genotypes. In early-senescing genotypes (PH6WC, PH4CV, and XY335), total chlorophyll and carotenoids ratios began to decline at 19 DAT and fell to approximately 50% of Ear+ levels by 25 DAT (Fig. S1a-b). This reduction was accompanied by a significant increase in anthocyanin accumulation—reaching over 4-fold higher than Ear+ at 25 DAT (Fig. S1c). In contrast, pigment content ratios remained largely stable in delay-senescing genotypes (Z58, C7-2, and ZD958) over the entire time course (Fig. S1). Collectively, these results demonstrate that maize genotypes exhibit contrasting response to ear removal, separating early-senescing and delay-senescing groups, and suggesting differential regulation of source–sink balance and senescence pathways among maize genotypes.

### Ear removal induced senescence is accompanied by a decline in photosynthetic capacity

**Figure 2:**
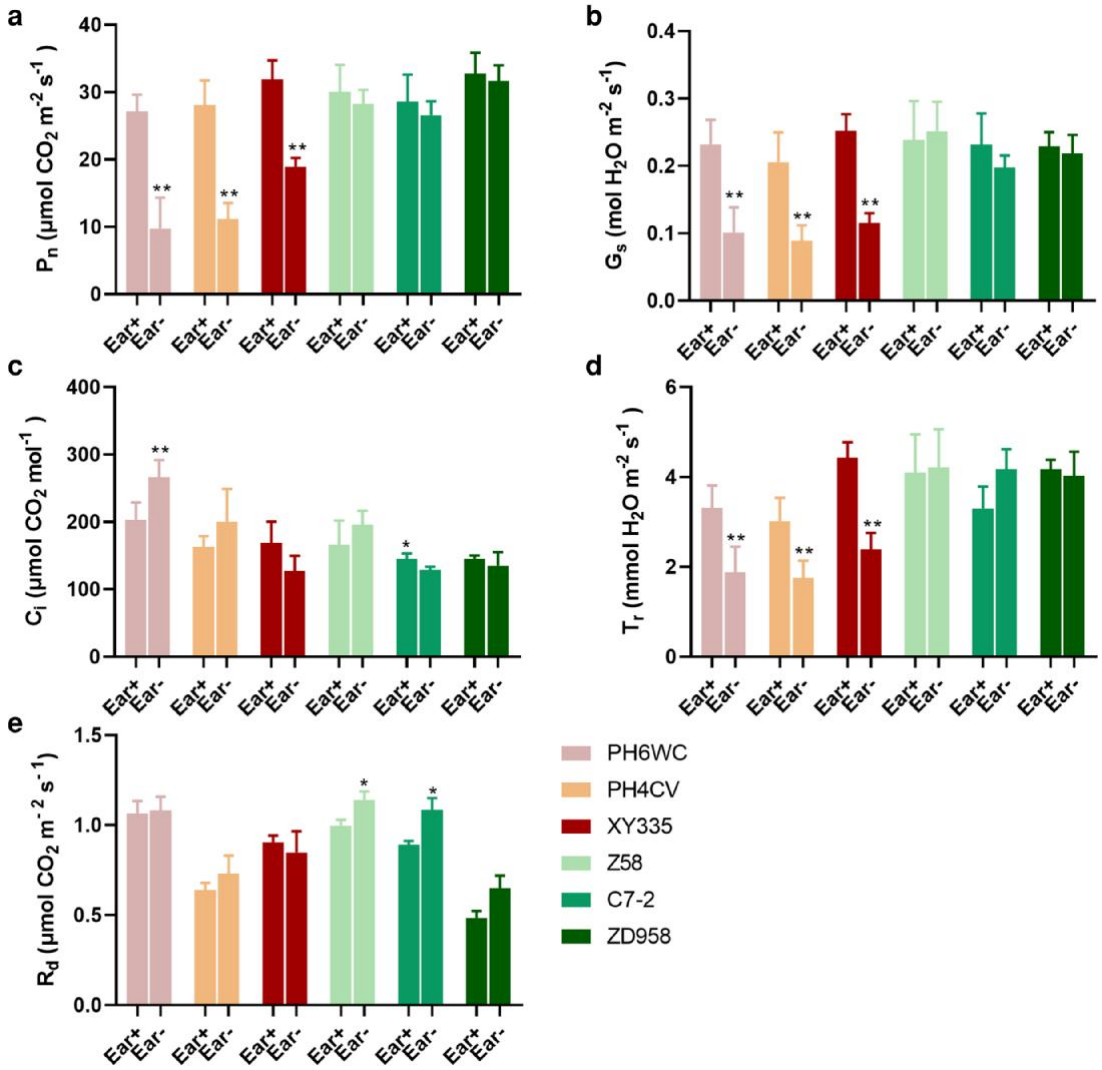
Gas exchange parameters of 6 maize genotypes at 25 DAT. (a) Net photosynthetic rate (P_n_); (b) stomatal conductance (G_s_); (c) intercellular CO_2_ concentration (C_i_); (d) transpiration rate (T_r_); (e) dark respiration rate (R_d_). Data are presented as means ± SEM (*n* ≥ 3). * and ** indicate statistically significant differences at *P* ≤ 0.05 and *P* ≤ 0.01, respectively, compared with Ear+ (Student's *t*-test). DAT, days after treatment.

To access the physiological consequences of pigment alterations on photosynthetic efficiency, gas exchange parameters were measured in ear leaves at 25 DAT. In the early-senescing genotypes (XY335, PH6WC, and PH4CV), ear removal significantly reduced net photosynthetic rate (P_n_) by 40.82%, 64.01%, and 60.26%, respectively, relative to the Ear+ (Fig. 2a). These reductions were accompanied by significant declines in stomatal conductance (G_s_) and transpiration rate (T_r_), indicating impaired photosynthetic capacity.

In contrast, delay-senescing genotypes (Z58, C7-2, and ZD958) displayed no significant differences in P_n_, G_s_, intercellular CO_2_ concentration (C_i_), or T_r_ between the Ear- and Ear+ treatments at 25 DAT (Fig. 2a-d), highlighting their resilience to sink removal. Interestingly, these genotypes exhibited elevated nighttime dark respiration rates under Ear- treatment relative to Ear+, while no such respiratory increase was detected in the early-senescing genotypes, implying a compensatory mechanism to maintain carbon balance when sink demand is reduced (Fig. 2e).

### Chloroplast ultrastructure is compromised during ear removal-induced senescence

**Figure 3:**
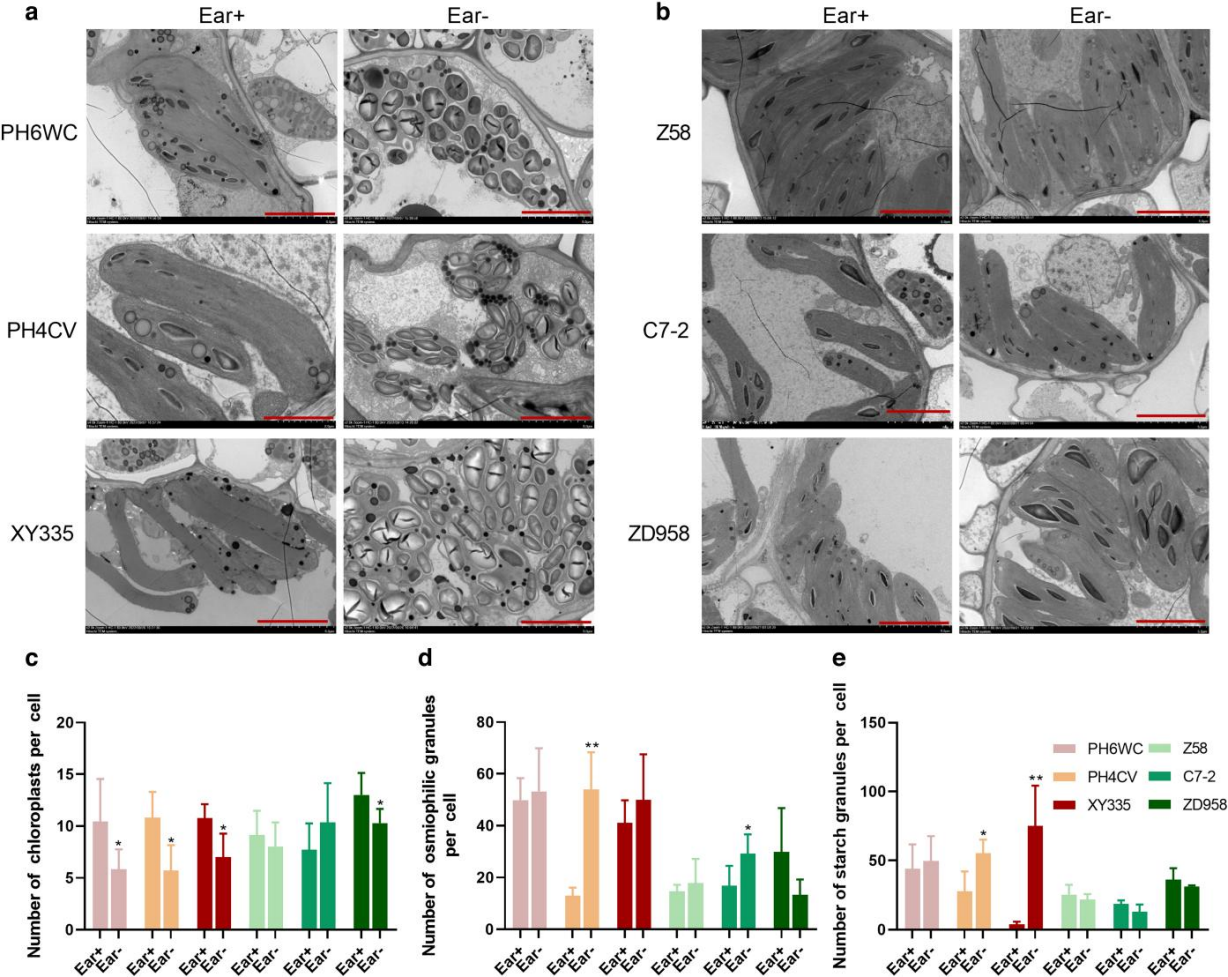
Chloroplast ultrastructure in bundle sheath cells (BSCs) of 6 maize genotypes at 25 DAT. (a-b) Transmission electron micrographs of BSC chloroplasts under Ear+ and Ear-. (c-e) Quantification of chloroplasts (c), osmophilic granules (d), and starch granules (e) per BSC. Data are presented as means ± SEM (*n* ≥ 10 independent sections obtained from 4 biological replicates). * and ** indicate statistically significant differences at *P* ≤ 0.05 and *P* ≤ 0.01, respectively, compared with Ear+ (Student's *t*-test). Red bar = 5.0 μm. DAT, days after treatment.

Given the central role of chloroplasts in photosynthesis and carbohydrate metabolism, transmission electron microscopy (TEM) was employed to assess chloroplast ultrastructure in both bundle sheath cells (BSCs) and mesophyll cells (MCs) at 25 DAT. In the early-senescing genotypes (PH6WC, PH4CV, and XY335), Ear- treated leaves exhibited pronounced ultrastructural damage in BSC chloroplasts, including significant chloroplast enlargement, distorted shape, dislocation toward the cell center, and excessive accumulation of starch granules. Severe structural degradation was evident, with disrupted chloroplast bilayer membrane, degraded stroma lamellae, and leakage of chloroplast inclusions into the cytoplasm (Fig. 3a). By contrast, BSC chloroplasts in the delay-senescing genotypes (Z58, C7-2, and ZD958) maintained typical ellipsoidal morphology with intact membrane and organized stroma lamellae structures under both Ear- and Ear+ conditions (Fig. 3b). Quantitative analyses further confirmed a reduction in the number of chloroplasts per BSC, along with increased starch granule and osmiophilic globule accumulation, specifically in the early-senescing genotypes (Fig. 3c-e).

Similar structural disruptions were also observed in MC chloroplasts of the early-senescing genotypes following ear removal. These chloroplasts became more spherical and showed thylakoid disorganization with reduced grana stacking, accompanied by marked increases in both starch grain size and osmiophilic globule abundance (Fig. S2a). In contrast, MC chloroplasts in delay-senescing genotypes and in all Ear+ controls maintained spindle-shaped morphology with well-organized thylakoid membranes, fewer and smaller starch granules, and lower osmiophilic globule abundance (Fig. S2a-e).

These results suggest that the sink removal triggers carbohydrate accumulation, which disrupts chloroplast structure and integrity, accelerating the senescence process in early-senescing genotypes. Importantly, despite these internal ultrastructural changes, no significant differences in overall leaf thickness or vascular bundle area were observed between Ear- and Ear+ treatments across 6 genotypes (Fig. S3), indicating that the observed senescence is primarily driven by cellular and organelle-level disruptions rather than changes in gross leaf anatomy.

### Excessive accumulation of photoassimilates acts as a trigger for ear removal-induced leaf senescence

**Figure 4:**
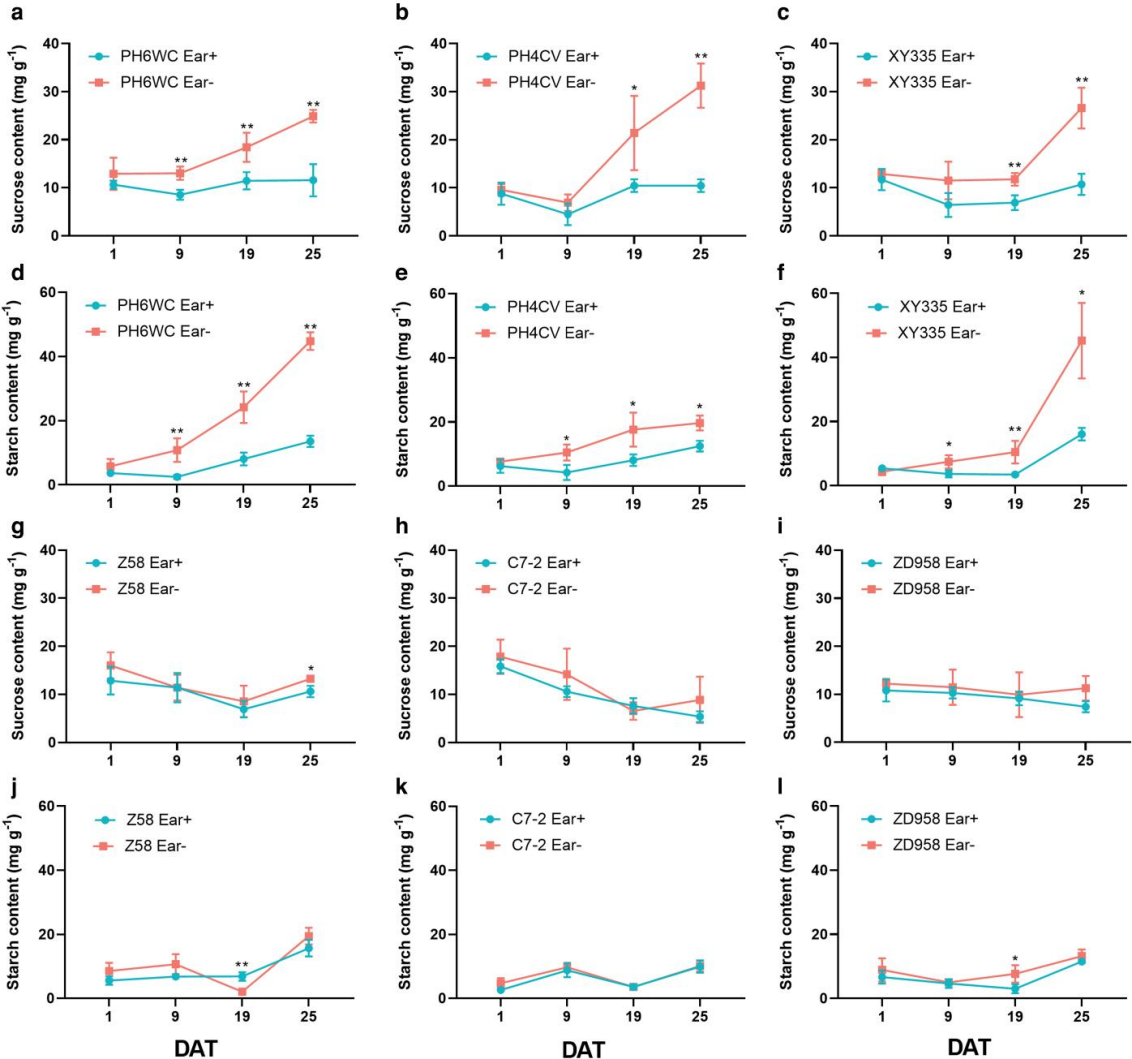
Sucrose and starch dynamics in ear leaves of 6 maize genotypes following Ear+ and Ear- treatments. (a-f) Sucrose (a-c) and starch (d-f) contents in ear leaves of PH6WC (a, d), PH4CV (b, e), and XY335 (c, f). (g-l) Sucrose (g-i) and starch (j-l) contents in ear leaves of Z58 (g, j), C7-2 (h, k), and ZD958 (i, l). Blue circles represent Ear+ treatment, and red squares represent Ear- treatment. The *x*-axis indicates days after treatment (DAT). Data are presented as means ± SEM (*n* ≥ 3). * and ** indicate statistically significant differences at *P* ≤ 0.05 and *P* ≤ 0.01, respectively, compared with Ear+ (Student's *t*-test).

To elucidate the role of carbohydrate accumulation in Ear- induced senescence, we monitored sugar dynamics in ear leaves following ear removal across contrasting maize genotypes (Fig. 4). In early-senescing lines (XY335, PH6WC, and PH4CV), Ear- treatment led to significant and progressive accumulation of sucrose and starch starting from 9 DAT, followed by increases in glucose and fructose levels at 25 DAT (Fig. S4). In contrast, delay-senescing genotypes (Z58, C7-2, and ZD958) showed minimal changes in sugar contents throughout the treatment period (Fig. 4g-l; Fig. S4).

**Figure 5:**
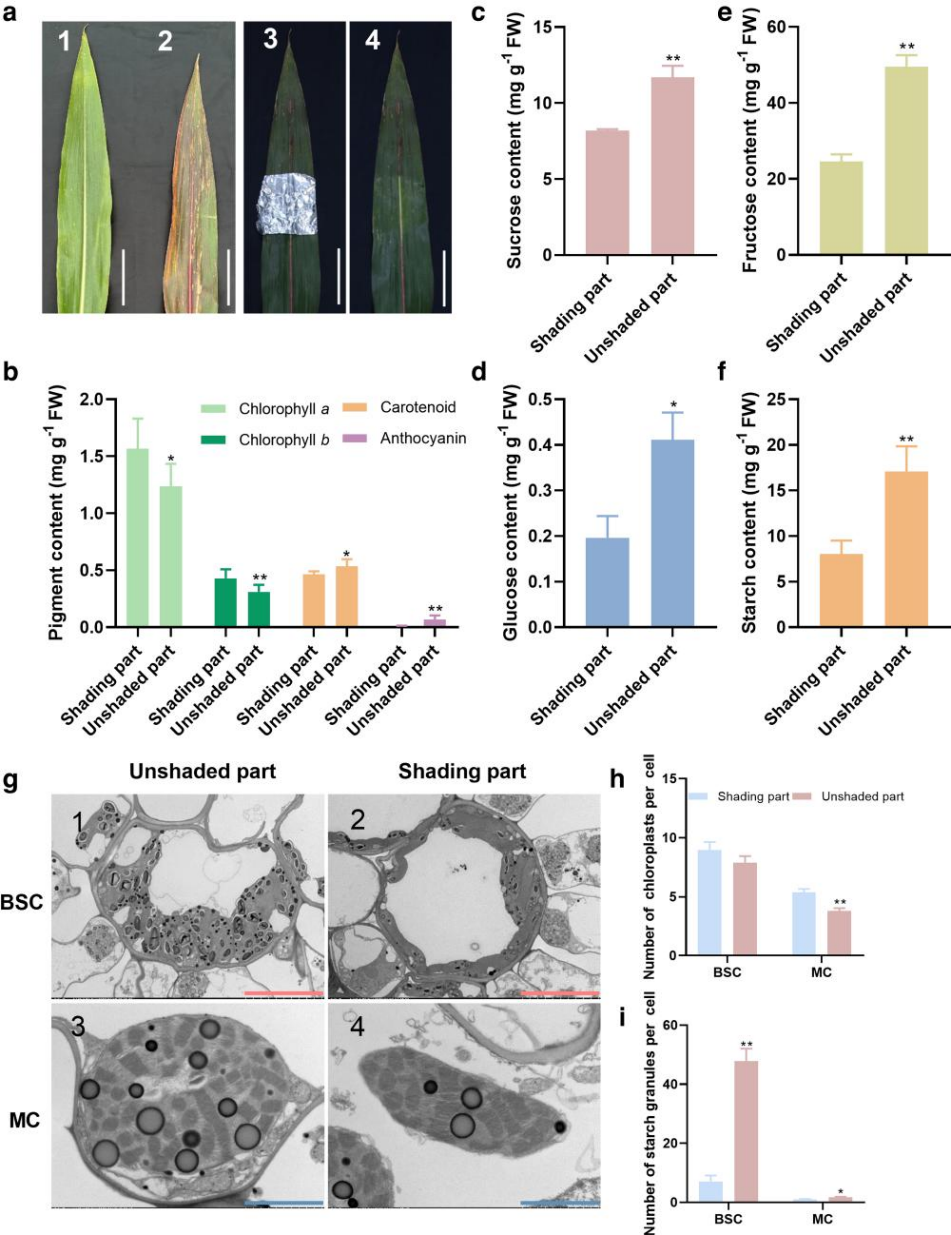
Leaf phenotypic and physiological changes in maize hybrid XY335 after Ear- and partial shading. (a) Leaf phenotypes change after 30 days of Ear+ (No.1), Ear- (No. 2), and (Ear-)+ shading (No. 3-shading, No. 4 same as No. 3 after removing the aluminum foil). (b) Chlorophyll *a*, *b*, carotenoids, and anthocyanins in the shaded part and unshaded parts. (c–f) Contents of sucrose (c), glucose (d), fructose (e), and starch (f). (g) TEM of BSCs (No.1-unshaded, No.2-shaded) and MCs (No.3-unshaded, No.4-shaded). (h–i) Number of chloroplasts (h) and starch granules (i) in BSCs and MCs. Data are presented as means ± SEM (*n* ≥ 5 for [b–f], and *n* ≥ 20 for [h–i]). * and ** indicate statistically significant differences at *P* ≤ 0.05 and *P* ≤ 0.01, respectively, compared with Ear+ (Student's *t*-test). Scale bars: red = 10 μm; blue = 2 μm.

Notably, the rise in sucrose content at 9 DAT preceded the sharp increase in anthocyanin levels at 25 DAT, suggesting that carbohydrate accumulation may act as a key upstream signal to activate anthocyanin biosynthesis and, ultimately, leaf senescence in susceptible genotypes (Fig. S1c). To further test this hypothesis, a shading experiment was conducted on the early-senescing genotype XY335 immediately after Ear- treatment. By 30 DAT, phenotypic differences were evident: shaded regions of the leaf retained green coloration and high chlorophyll contents, while unshaded regions exhibited pronounced reddening, anthocyanin accumulation, and senescence symptoms (Fig. 5a-b).

Physiological and ultrastructural analyses demonstrated that unshaded regions exhibited elevated levels of sugar and starch, accompanied by enlarged and structurally compromised chloroplasts. These chloroplasts displayed disrupted thylakoid membranes, incomplete stroma lamellae, and an accumulation of excessive starch granules in both BSCs and MCs. In contrast, shaded regions maintained lower sugar contents and intact chloroplast ultrastructure (Fig. 5c-i), which was associated with delayed leaf senescence. These findings further highlight carbohydrate overaccumulation as a central regulator of sink-removal-induced senescence.

### Ear removal modifies biomass allocation differently in early- and delay-senescing maize genotypes

**Figure 6:**
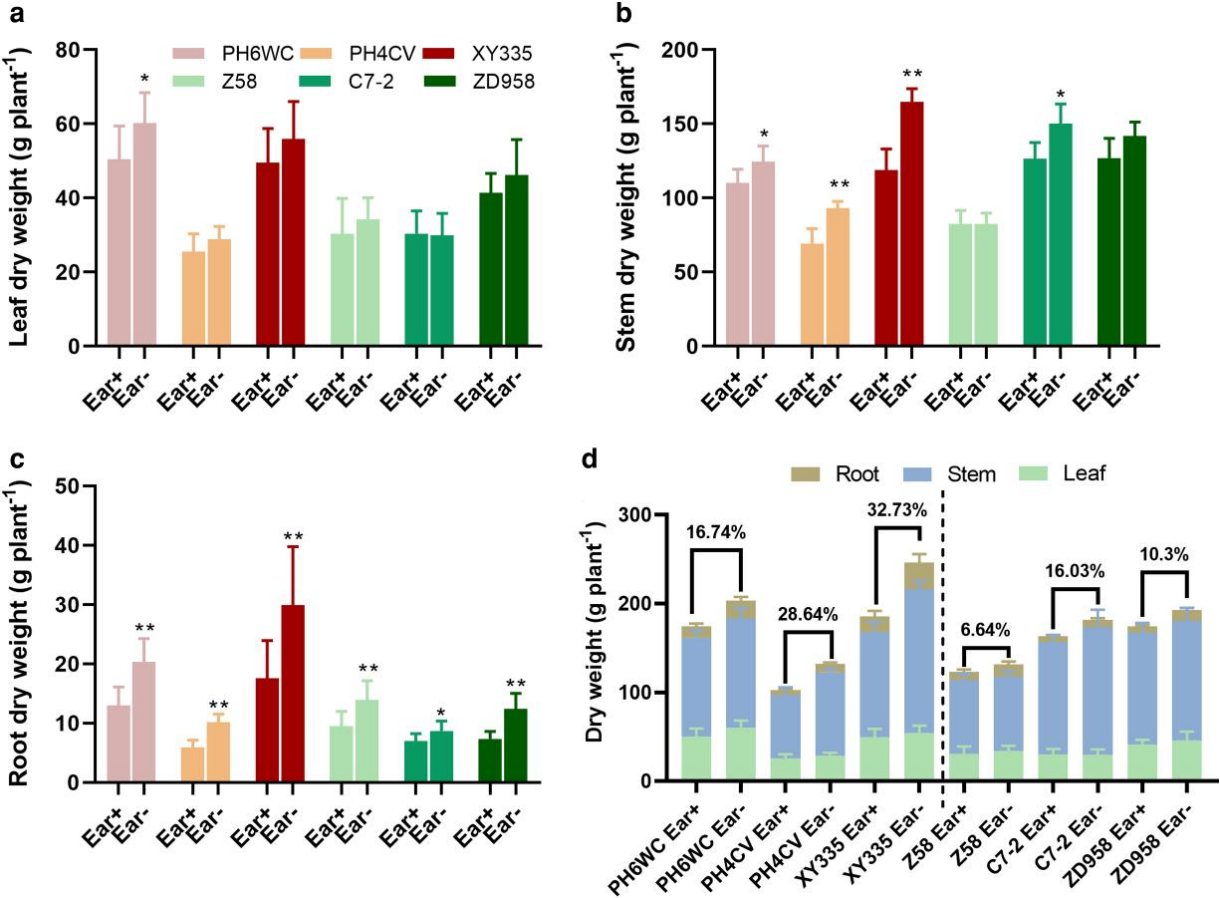
Biomass accumulation and distribution in 6 maize genotypes at 30 DAT under Ear+ and Ear- treatments. (a–d) Dry weight of leaves (a), stem (b), root (c), and total biomass in roots, stems, and leaves (d). Data are presented as means ± SEM (*n* = 10). * and ** indicate statistically significant differences at *P* ≤ 0.05 and *P* ≤ 0.01, respectively, compared with Ear+ (Student's *t*-test). DAT, days after treatment.

To further explore the physiological consequences of Ear- induced senescence, we evaluated the biomass accumulation and partitioning among leaves, stems, and roots during the progression of leaf senescence following Ear- treatment. Leaf dry weight showed a significant increase only in PH6WC, whereas the other 5 genotypes displayed no significant differences compared to the Ear+ controls (Fig. 6a). Stem dry weights were increased significantly in PH6WC, PH4CV, and XY335; while among the delay-senescing genotypes, only C7-2 exhibited a significantly higher stem biomass relative to Ear+ group (Fig. 6b). Root dry weights were consistently elevated across all genotypes under Ear- treatment compared to their respective Ear+ groups (Fig. 6c).

Overall, total biomass (sum of leaves, stems, and roots) was enhanced across all genotypes in response to Ear- treatment. However, the magnitude of increase was greater in early-senescing genotypes (16.74%–32.73%) than in delay-senescing genotypes (6.64%–16.03%) (Fig. 6d). These results suggest that Ear- promotes biomass redistribution, particularly to roots and stems, with early-senescing genotypes showing more pronounced shifts in source–sink dynamics.

### Abscisic acid accumulation does not drive ear removal-induced leaf senescence

ABA is widely recognized as a senescence-promoting hormone, particularly under stress conditions. To evaluate its role in Ear- induced senescence, we monitored dynamic changes in ABA content in maize leaves following Ear- treatment. At 1 DAT, ABA levels were significantly elevated in 4 genotypes (PH4CV, Z58, C7-2, and ZD958) compared with Ear+ controls, with this difference persisting until 9 DAT in PH4CV, C7-2, and ZD958 (Fig. S5b, d, e, f). Furthermore, PH6WC also exhibited increased ABA levels by 9 DAT (Fig. S5a). By 25 DAT, all 6 genotypes accumulated significantly higher ABA levels in response to the Ear- treatment (Fig. S5).

However, the delay-senescing genotypes consistently showed higher ABA accumulation, despite displaying delayed or reduced senescence phenotypes. This discrepancy suggests that although ABA level is elevated during Ear- induced senescence, its accumulation may be a downstream consequence rather than a cause of the process. Therefore, ABA is likely not the primary regulator driving Ear- induced leaf senescence in maize.

### Integrated metabolomic and transcriptomic analyses reveal candidate genes regulating source–sink dynamics in maize

To elucidate the molecular mechanisms underlying source–sink imbalance caused by ear removal, we performed time-course metabolomic and transcriptomic analyses on parental inbred lines representing early-senescing (PH6WC, PH4CV) and delay-senescing (Z58, C7-2) genotypes. Leaf samples were collected at 9, 19, and 25 DAT under Ear+ and Ear- conditions, based on physiological observations and previous studies, to capture early molecular divergence prior to visible senescence (except 9 DAT for metabolomics).

Through untargeted metabolomic profiling, we detected 775 differential metabolites, including primary and secondary metabolites, across 19 and 25 DAT (Table S1-S3). Principal component analysis of metabolite profiles indicated that genotypes were the primary determinant of metabolic variation, with species-specific signatures prevailing over treatment or time-point effects (Fig. S6a). This trend was further reflected in genotype-specific differences in metabolite abundance (Fig. S6b). Metabolite profiling revealed an overall increase in sugar metabolism following Ear- treatment, particularly in early-senescing genotypes. Additionally, phosphoenolpyruvate (PEP) metabolism pathways were notably upregulated in these lines. In contrast, Ear- treatment in delay-senescing genotypes led to a reduction in tricarboxylic acid (TCA) cycle activity, accompanied by accumulation of aspartate family amino acids, suggesting a compensatory shift in energy metabolism (Fig. S6c). These results highlight distinct metabolic reprogramming between early-senescing and delay-senescing genotypes in response to Ear- induced source–sink imbalance.

**Figure 7:**
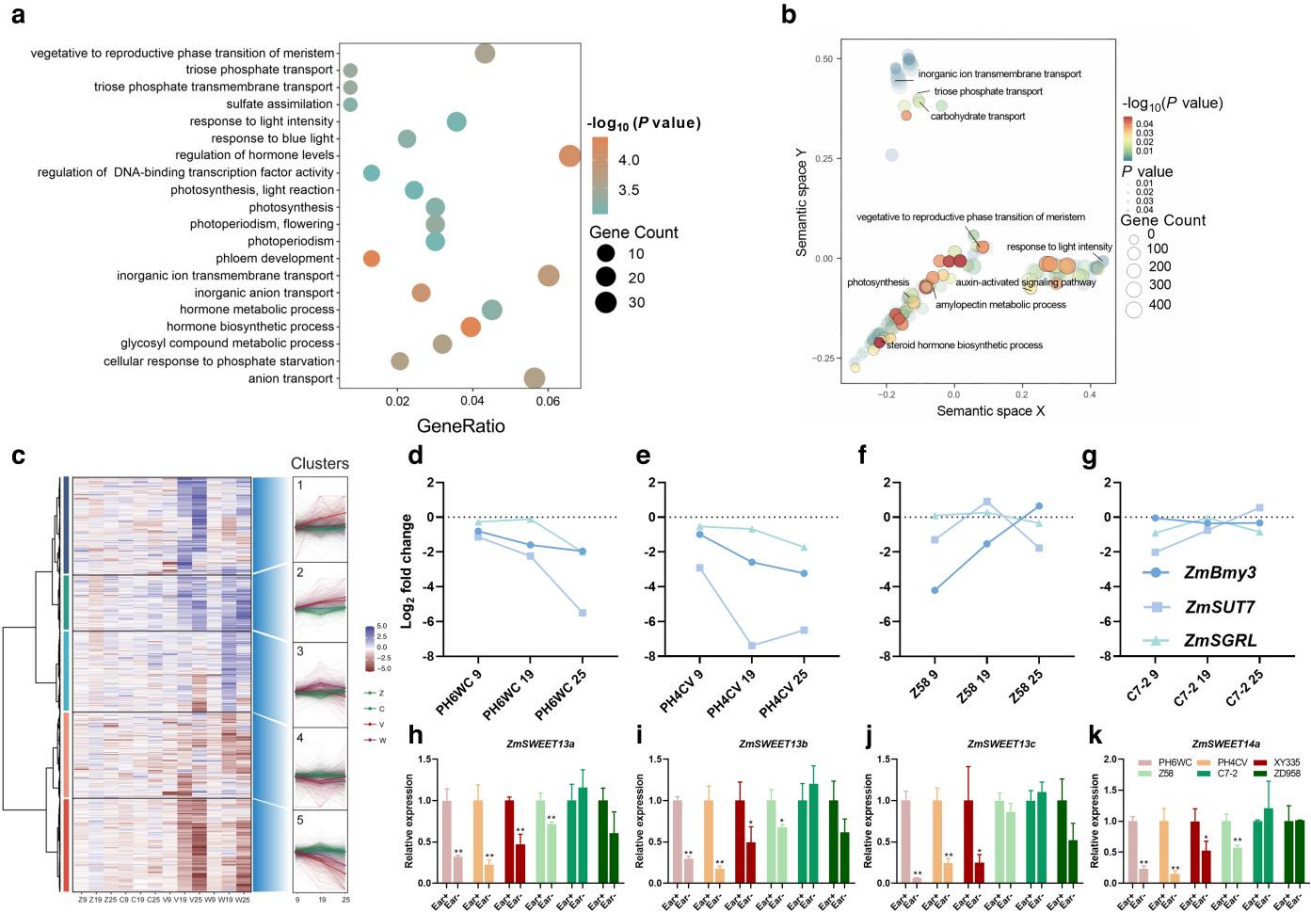
Transcriptomic analysis of 4 inbred lines PH6WC (W), PH4CV (V), Z58 (Z), and C7-2 (C). (a) GO enrichment analysis. (b) Functional categorization of selected gene sets. (c) Identification and clustering of 1,673 senescence-specific DEGs (at 19 DAT), grouped by expression patterns across genotypes. (d-g) Transcriptional changes of 3 key candidate genes in the 4 inbred lines PH6WC (d), PH4CV (e), Z58 (f), and C7-2 (g), presented as log_2_ fold change (log_2_ FC) of Ear- relative to Ear+. The *x*-axis indicates days after treatment (DAT). (h-k) Relative expression of *ZmSWEET13a* (h), *ZmSWEET13b* (i), *ZmSWEET13c* (j), and *ZmSWEET14a* (k) at 25 DAT under Ear+ and Ear- treatments. Data are presented as means ± SEM (*n* = 3 for [a-g]; *n* ≥ 3 for [h-k]). * and ** indicate statistically significant differences at *P* ≤ 0.05 and *P* ≤ 0.01, respectively, compared with Ear+ (Student's *t*-test). DAT, days after treatment. DEG, differentially expressed gene.

Transcriptomic profiling revealed a progressive increase in the number of differentially expressed genes (DEGs) over time, with 1,317 DEGs identified at 9 DAT, 5,934 at 19 DAT, and 17,325 at 25 DAT (Fig. S7a; Table S4-S7). Considering that senescence-related phenotypes became evident at 19 DAT, subsequent analyses focused on this time point. A set of 1,673 DEGs specifically associated with senescence responses in the early-senescence genotypes (PH6WC and PH4CV) (Fig. S7b; Table S8) was enriched in pathways related to carbon metabolism, sugar and starch metabolism, membrane transport, and photosynthesis by Gene Ontology (GO) enrichment analysis (Fig. 7a-b).

K-means clustering analysis grouped the 1,673 DEGs into 5 distinct expression patterns (Fig. 7c). Among these, we focused on the consistently upregulated and downregulated clusters, as they likely represent core transcriptional responses to Ear- induced senescence (Fig. S8-S13). Kyoto Encyclopedia of Genes and Genomes (KEGG) enrichment analysis revealed that upregulated genes (Cluster 2) in early-senescing genotypes were strongly associated with anthocyanin biosynthesis, flavonoid metabolism, starch and sucrose metabolism, amino acid biosynthesis, and fatty acid degradation pathways (Fig. S9). In contrast, downregulated genes (Cluster 5) were predominantly enriched in photosynthesis-related pathways, including antenna protein biosynthesis (Fig. S12). These contrasting expression patterns were consistent with the physiological observations: activation of secondary metabolism and carbon reallocation, together with suppression of photosynthetic processes, underpin the anthocyanin accumulation, chlorophyll decline, and reduced photosynthetic capacity observed in senescing leaves.

Further analysis identified several candidate regulatory genes with distinct expression patterns between early- and delay-senescing genotypes across time points (Fig. S14). Among these, 3 key DEGs were identified: *ZmSUT7* (Zm00001eb402200), involved in sucrose transport; *ZmBmy3* (Zm00001eb016870), involved in starch degradation; and *ZmSGRL* (Zm00001eb076680), involved in chlorophyll breakdown. These genes were progressively downregulated in early-senescing leaves, but remained relatively stable in delay-senescing genotypes (Fig. 7d-g). Similarly, SWEET family members (*ZmSWEET13a/b/c*, *ZmSWEET14a*) showed marked downregulation in early-senescing genotypes, but only moderate decreases in delay-senescing lines (Fig. 7h-k). These transcriptional changes indicated that impaired sugar export and disrupted starch turnover play central roles in mediating Ear- induced leaf senescence.

To further explore regulatory networks associated with senescence following ear removal, weighted gene coexpression network analysis (WGCNA) was performed on 18,442 DEGs. A soft threshold power of 16 was selected for scale-free network construction, yielding 33 coexpression modules (Fig. S15a). Module–trait association analysis revealed that the MEpink and MEturquoise modules were significantly positively correlated with delayed senescence and negatively correlated with early-senescing after Ear- treatment (Fig. S15b-c). KEGG and GO enrichment analyses of hub genes (|MM| > 0.8, |GS| > 0.2) revealed that the MEturquoise module was strongly enriched in photosynthesis-related pathways (Fig. S16), while the MEpink module was enriched in pathways associated with cell wall biosynthesis, cellulose degradation, and cellulose synthesis (Fig. S17). Hub genes related to the senescence degree were identified based on weight values and intramodular connectivity (weight > 0.2 in MEturquoise; weight > 0.15 in MEpink). Among these, *Zm00001eb325370*, *Zm00001eb016250*, *Zm00001eb353810*, *Zm00001eb146750*, and *Zm00001eb208970* showed high connectivity and were implicated in carbohydrate metabolism and amino acid metabolic processes (Fig. S16, S17).

### Discussion

Previous studies have shown that sink limitation in maize—whether induced by pollination prevention or ear removal—drives systemic physiological changes, including sugar overaccumulation, stress signaling activation, and premature leaf senescence, with substantial genotype-dependent variation (Ceppi et al. 1987; Kumar et al. 2019, 2023). These contrasting senescence responses reflect the intrinsically complex regulation of leaf aging, which integrates sugar metabolism, ABA and ROS signaling, and secondary metabolism. ABA and ROS have been proposed as early mediators of weakened sink demand, converging to trigger endoplasmic reticulum (ER) stress and lead to programmed cell death, further initiating senescence (Kumar et al. 2023). Meanwhile, the capacity to redirect accumulated nonstructural carbohydrates into secondary sinks is considered a major determinant of whether leaves senesce rapidly or maintain a stay-green phenotype (Kumar et al. 2019). Central to this process is T6P, a sugar-signaling metabolite that modulates senescence via SnRK1 inhibition, thereby coupling carbohydrate status to developmental aging (Kumar et al. 2019).

Building on these established foundations, our multiyear, multigenotype analyses refine and extend current models of sink-removal-induced senescence by identifying additional regulatory layers. We show that starch overaccumulation and associated chloroplast disruption represent a decisive trigger of senescence; that ABA functions primarily as a secondary stress responder rather than a primary signal; and the enhanced dark respiration in delay-senescing genotypes provides a previously unrecognized metabolic strategy to mitigate carbon overload.

Although ABA is widely regarded as a major regulator of stress-induced senescence (Quiles et al. 1995; Zhao et al. 2022), previous work has further linked ABA accumulation under sink limitation to ROS-mediated ER stress and premature senescence in maize (Kumar et al. 2023). Consistent with these findings, we observed elevated ABA levels following sink removal, but ABA alone does not determine differential senescence timing. ABA increased in both early- and delay-senescing genotypes—and even earlier in some delay-senescing lines (Fig. S5)—indicating that its accumulation is a general stress response rather than a senescence trigger. Differences from prior studies—such as divergent ABA dynamics in B73 and Mo17 (Kumar et al. 2023)—likely reflect genotypic variation and methodological differences, as ear removal introduces mechanical injury, whereas nonpollination preserves the cob as a transient sink. Consistent with this, KEGG analysis showed no enrichment of ABA-related pathways among senescence-associated DEGs (Fig. S13). These findings collectively redefine ABA as a secondary, modulatory regulator rather than a determinative driver of sink-removal-induced senescence.

In contrast, carbohydrate signaling emerged as a dominant driver of senescence under source–sink imbalance. Ear removal induced significant sucrose accumulation beginning at 9 DAT, especially in early-senescing genotypes (Fig. 4), accompanied by downregulation of key sucrose transporter genes (*ZmSWEET13a/b/c* and *ZmSWEET14a*) required for apoplastic phloem loading (Chen et al. 2012; Bezrutczyk et al. 2018). Impaired sugar export likely intensified carbohydrate overload and activated hexokinase (HXK1)-mediated sugar-signaling pathways (Smeekens et al. 2010). Elevated sugar also promoted antioxidant flavonoid and anthocyanin biosynthesis—a conserved sugar-response pathway (LaFountain and Yuan 2021; Ma et al. 2021; Sunil and Shetty 2022)—supported by the induction of flavonoid biosynthesis genes (Fig. 1e; Fig. S8) and further verified by shading experiments (Fig. 5a–f). These findings support the view that excessive carbohydrate retention in leaves is a central driver of sink-removal-induced senescence.

A key finding of this study is the identification of starch, rather than soluble sugars alone, as a decisive mediator of premature senescence. Early-senescing genotypes accumulated excessive starch within chloroplasts (Fig. 3; Fig. S2), coincident with repression of the starch degradation gene *ZmBmy3* and feedback inhibition of photosynthesis (Goldschmidt and Huber 1992). Critically, starch overaccumulation severely disrupted chloroplast ultrastructure, leading to chloroplast swelling, thylakoid disorganization, and reduced protein content (Fig. S18). Although similar phenotypes have been reported in starch metabolism mutants (Zhu et al. 2020; Qin et al. 2022), our work provides evidence that starch-induced chloroplast damage acts as a proximate trigger of senescence following sink removal in maize, functioning alongside, but distinct from, sugar- and ROS-mediated pathways (Fig. 4-5).

In contrast, delay-senescing genotypes exhibited markedly greater capacity to buffer carbon overload. Although both early- and delay-senescing genotypes redirected sugars into secondary sinks such as roots and stems (Fig. 6), consistent with prior studies (Kumar et al. 2019; Sekhon et al. 2019), this process occurred in both groups and therefore does not fully explain differences in senescence timing. Instead, our comparative analyses indicate that delayed senescence is additionally supported by a respiration-based compensatory mechanism. Delay-senescing genotypes exhibited higher dark respiration while maintaining photosynthetic capacity (Fig. 2a, 2e), enabling more effective consumption of excess carbon and preventing starch overaccumulation. This respiratory compensation helped preserve chloroplast structure (Ren et al. 2024) and sustained functional greenness (Fig. 4g–l). By prolonging photosynthetic duration, delayed senescence enables continuous allocation of assimilates to secondary sinks (eg late-developing kernels) or storage tissues such as stems and roots, reflecting positive metabolic flexibility and adaptive value under both sink-limited and normal conditions. This mechanism contributes to enhanced biomass stability and may benefit silage-type maize production.

Integrated transcriptomic and metabolomic analyses further revealed distinct metabolic architectures underlying these divergent senescence strategies. Consistent with previous studies showing that early-senescing lines accumulate more primary metabolites—such as sugar alcohols (eg mannitol, erythritol) and amino acids including phenylalanine and arginine—while stay-green lines preferentially accumulate secondary metabolites, such as phenylpropanoids and flavonoids (eg naringenin chalcone, eriodictyol) (Brar et al. 2025), our results confirm and extend these patterns under sink limitation. Following ear removal, early-senescing genotypes exhibited carbon-metabolic rigidity, accumulating TCA-cycle and PEP-pathway intermediates together with elevated sucrose and starch (Figs. 4a–f, S6). In contrast, delay-senescing genotypes maintained carbon homeostasis and redirected carbon into the aspartate family amino acids (lysine, isoleucine, methionine), reflecting a shift that competes with pyruvate entry into the TCA cycle (Galili 2011). This reallocation reinforces carbon–nitrogen coupling, prevents accumulation of central carbon intermediates, and contributes to metabolic stability after sink disruption (Wu et al. 2018). Together with elevated respiration, these adjustments define the metabolic flexibility of delay-senescing genotypes.

Coexpression network analysis connected these physiological and metabolic differences to distinct regulatory modules. Delay-senescing genotypes showed enrichment of cell wall biosynthesis pathways and hub genes involved in carbohydrate and amino acid metabolism (Fig. S15, S16, S17), consistent with the proposed roles of carbon partitioning into structural sinks for promoting stay-green traits in maize (Sekhon et al. 2019). Notably, key senescence-related genes—including *ZmSUT7*, *ZmBmy3*, *ZmSGRL*, and *ZmSWEETs*—showed stable or increased expression in delay-senescing lines but were strongly repressed in early-senescing genotypes (Fig. 7d–k). These contrasting patterns align with observed differences in sucrose export, starch degradation (Fig. 4a–f), and chlorophyll retention (Fig. 1b–d; Kumar et al. 2023), identifying these genes as strong candidates for functional validation of stay-green mechanisms in maize.

**Figure 8:**
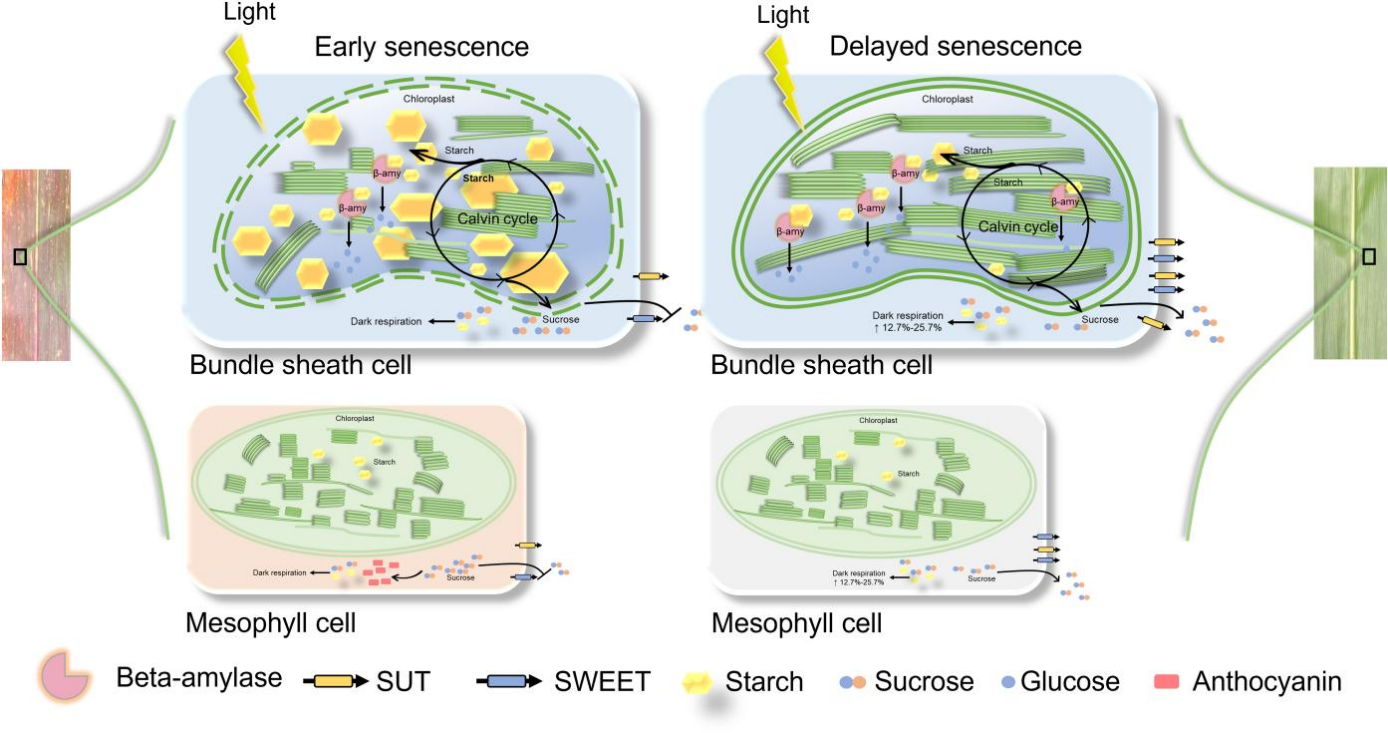
Proposed model of leaf phenotypic changes in response to ear removal in different maize genotypes. After Ear- treatment, the 6 maize genotypes exhibited distinct leaf senescence responses: PH6WC, PH4CV, and XY335 displayed premature leaf senescence, whereas Z58, C7-2, and ZD958 maintained delayed senescence. In early-senescing genotypes, Ear- induced significant downregulation of sugar transport (*ZmSUT*, *ZmSWEETs*) and starch metabolism (*ZmBmy3*) genes, leading to excessive starch accumulation in bundle sheath cells and sucrose buildup in mesophyll cells. This metabolic imbalance promoted anthocyanin accumulation (leaf reddening), starch granule hypertrophy, thylakoid degeneration, and eventual chloroplast lysis, collectively accelerating senescence. By contrast, delay-senescing genotypes maintained stable expression of transport and metabolic genes, supporting efficient sugar export and respiratory carbon consumption, preventing sugar overload, preserving chloroplast integrity, and sustaining normal leaf function.

In conclusion, while previous studies identified several responses to sink removal, our multiyear, multiomics analyses reveal mechanistic distinctions between early- and delay-senescing maize genotypes under sink limitation. Early-senescing lines (XY335, PH6WC, and PH4CV) exhibited impaired sucrose export, starch overaccumulation, chloroplast damage, and accelerated senescence; whereas delay-senescing lines (ZD958, Z58, and C7-2) maintained metabolic homeostasis through enhanced respiration and stable regulation of key transport and metabolic genes (Fig. 8). Together, these findings (i) refine ABA's role to a secondary regulator, (ii) identify starch-induced chloroplast damage as a key initiator of premature senescence, (iii) establish respiration-based metabolic compensation as a determinant of delayed senescence, and (iv) highlight candidate regulatory genes involved in source–sink balance. Although focused on 2 widely cultivated hybrids, ZD958 and XY335, and their parental lines, and thus may not fully capture the diversity of senescence response across all maize genotypes, our work broadens current source–sink regulatory models and provides promising targets for improving photosynthetic longevity and yield stability in maize.

### Accession numbers

The sequence data for the major genes discussed in this paper are available in the GenBank/EMBL databases under the following accession numbers: *ZmSUT7* (NM_001308124.1), *ZmBmy3* (XM_020546376.1), *ZmSGRL* (NM_001137437.1), *ZmSWEET13a* (NM_001155615.2), *ZmSWEET13b* (NM_001148182.1), *ZmSWEET13c* (NM_001147634.1), *ZmSWEET14a* (NM_001139364.1).

## Supplementary Material

kiag190_Supplementary_Data

## Data Availability

The data generated in this study are included in this article and the online supplementary material.
