# Supplementary material for: Genotypic variation in maize leaf senescence after ear removal is caused by excess accumulation of photosynthates
Source: Plant Physiol. 2026 Apr 8;201(1):kiag190. doi: 10.1093/plphys/kiag190 (PMC13189543; doi:10.1093/plphys/kiag190)
Supplement: kiag190_Supplementary_Data [file kiag190_supplementary_data.zip › Supplementary Figure -20260326.pdf]

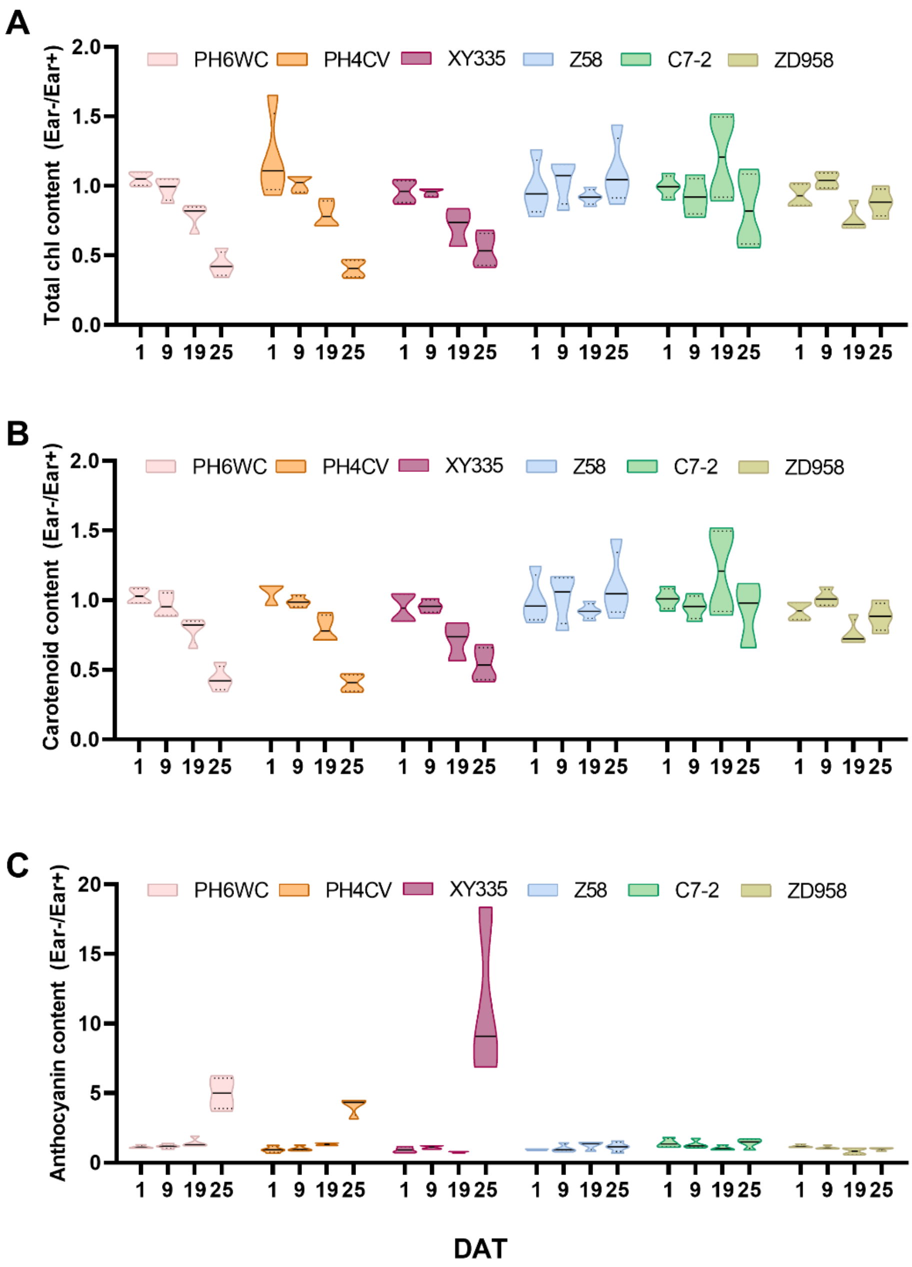

Supplementary Figure S1. Ratio of pigment content in six maize genotypes after ear removal (Ear-) treatment.

(A-C) Ratio of Ear- to Ear+ for total chlorophyll (A), carotenoids (B), and anthocyanins (C) in ear leaves over time. The x-axis represents days after treatment (DAT), and the y-axis represents the Ear-/Ear+ ratio ($n \geq 3$). The solid line in the center of the violin box represents the median; the thin dashed line at the top of the box represents the third quartile (Q3), and the thin dashed line at the bottom represents the first quartile (Q1). The width of the violin box reflects the density estimate of the data distribution.

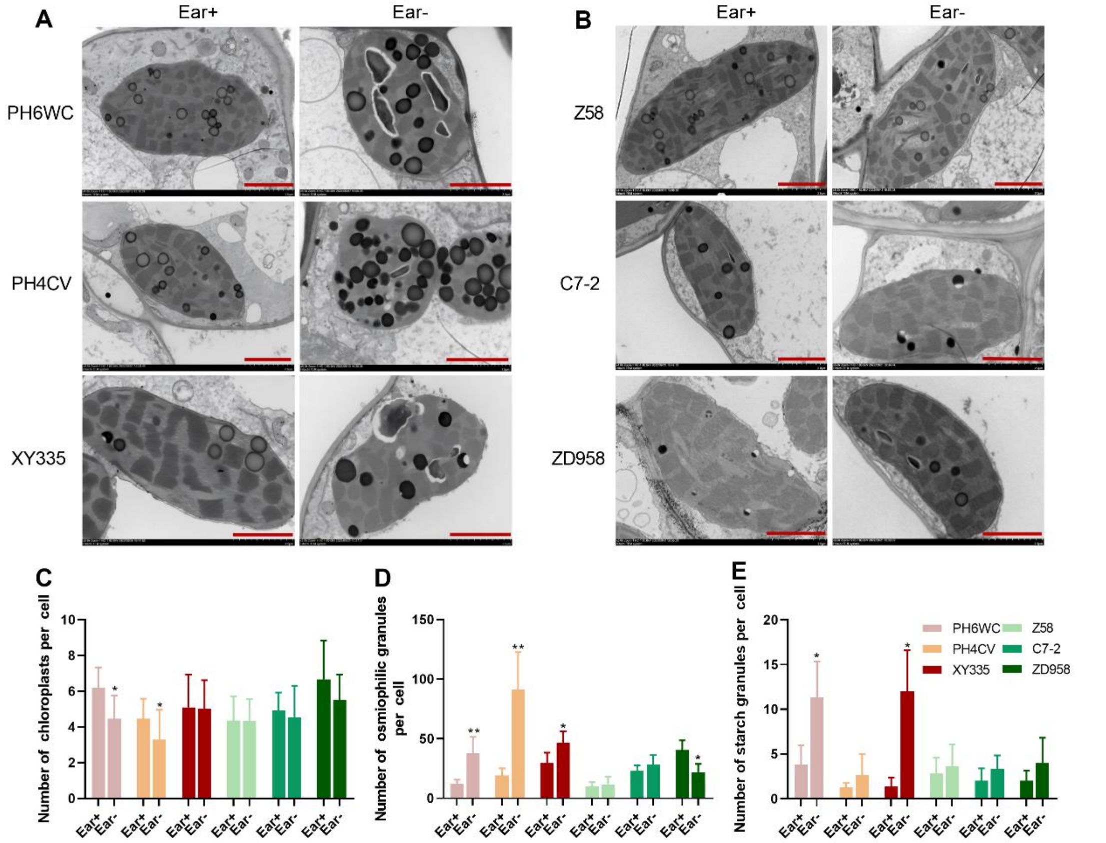

Supplementary Figure S2. Chloroplast ultrastructure in mesophyll cells (MCs) of six maize genotypes at 25 DAT.

(A-B) Transmission electron micrographs of MC chloroplasts under Ear+ and Ear-. (C-E) Quantification of chloroplasts (C), osmophilic granules (D), and starch granules (E) per MC. Data are presented as means ± SEM ($n \geq 10$ independent sections obtained from four biological replicates). * and ** indicate statistically significant differences at $P \leq 0.05$ and $P \leq 0.01$, respectively, compared with Ear+ (Student's *t*-test). Scale bar = 2.0 μm. DAT, days after treatment.

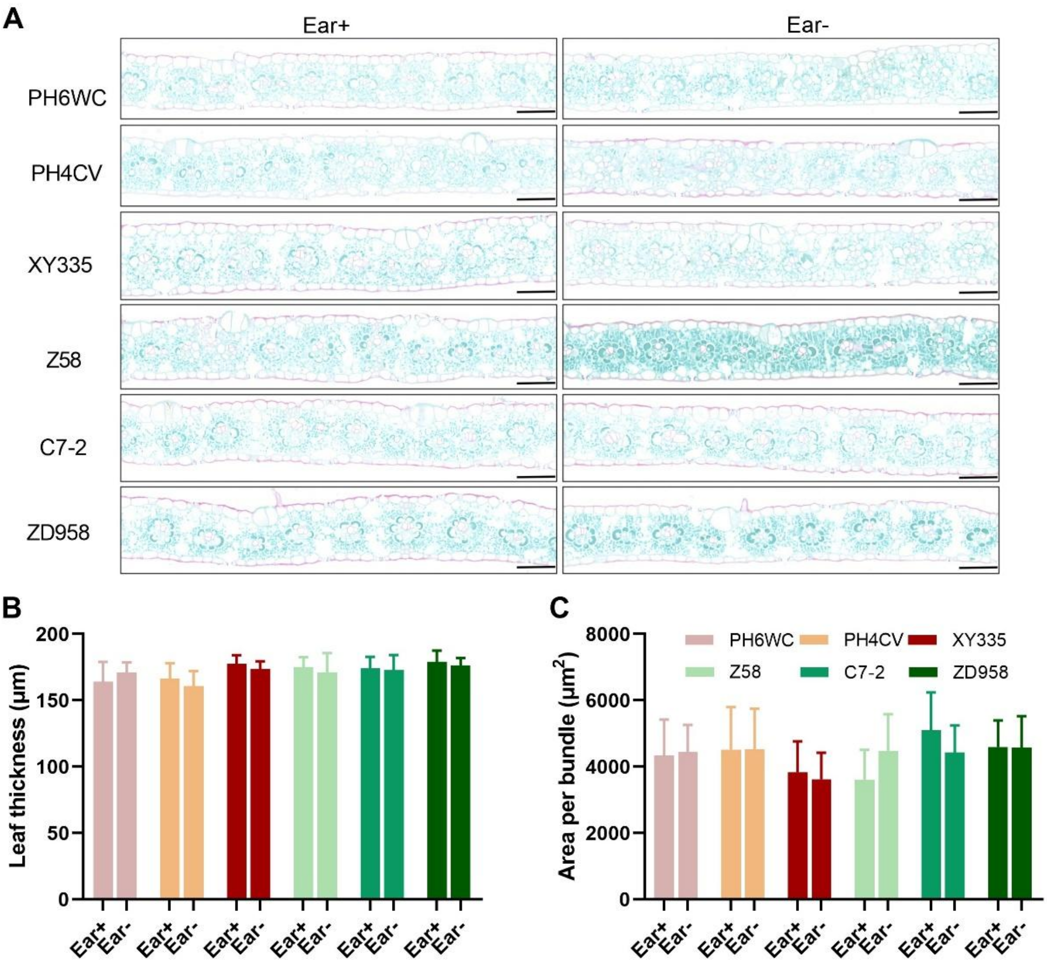

Supplementary Figure S3. Microscopic analysis of leaf cross-section in six maize genotypes at 25 DAT under Ear+ and Ear- treatments.

(A) Representative cross-sectional leaf images. Scale bar = 100 μm. (B) Blade thickness. (C) Area of small vascular bundles. Data are presented as means ± SEM (*n* = 20 sections for (B), and 60 sections for (C) obtained from four biological replicates). DAT, days after treatment.

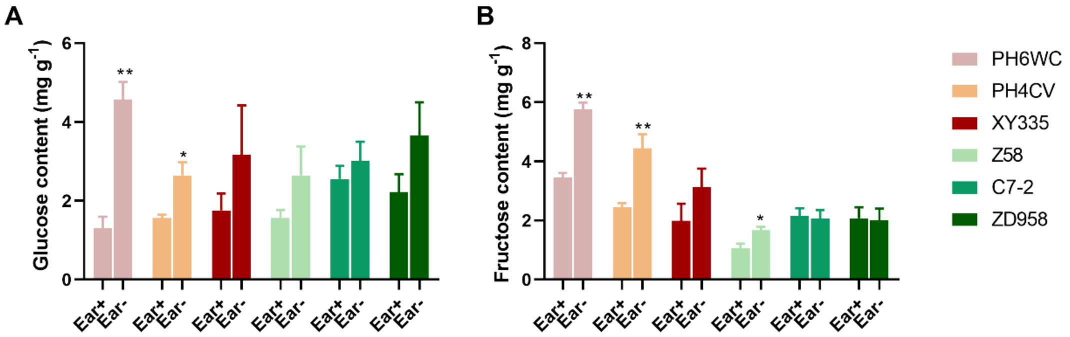

Supplementary Figure S4. Glucose and fructose content in six maize genotypes at 25 DAT under Ear+ and Ear- treatments.

(A) Glucose and (B) fructose content in ear leaves. Data are presented as means ± SEM ($n \geq 3$). * and ** indicate statistically significant differences at $P \leq 0.05$ and $P \leq 0.01$, respectively, compared with Ear+ (Student's *t*-test). DAT, days after treatment.

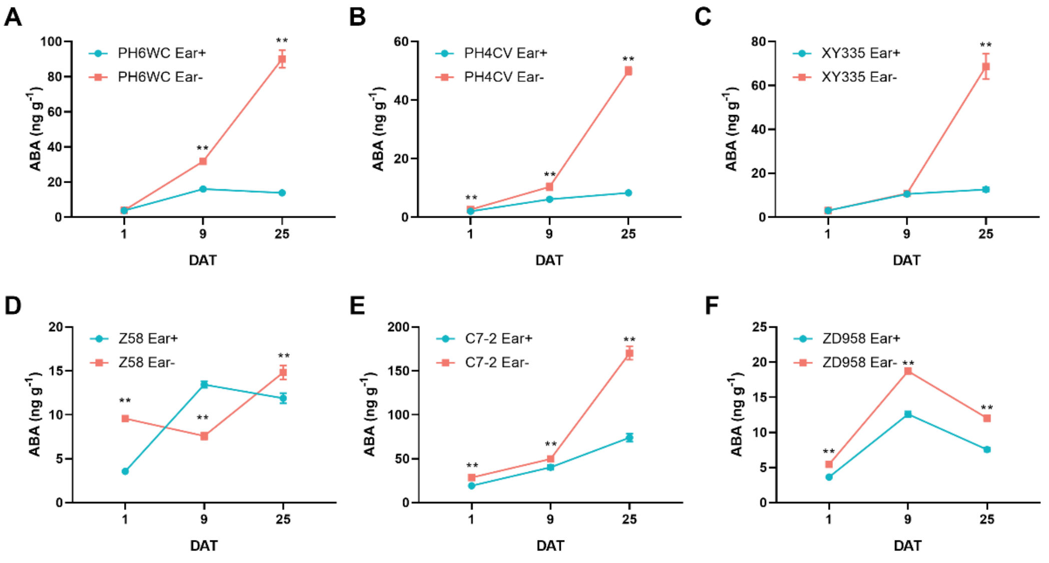

Supplementary Figure S5. Abscisic acid (ABA) content in ear leaves of six maize genotypes.

(A-F) ABA contents in ear leaves of PH6WC (A), PH4CV (B,) and XY335 (C), Z58 (D), C7-2 (E) and ZD958 (F). Blue circles represent Ear+ treatment, and red squares represent Ear- treatment. The x-axis indicates days after treatment (DAT). Data are presented as means ± SEM (n = 3). ** indicates statistically significant differences compared with Ear+ at $P \leq 0.01$ (Student's t-test).

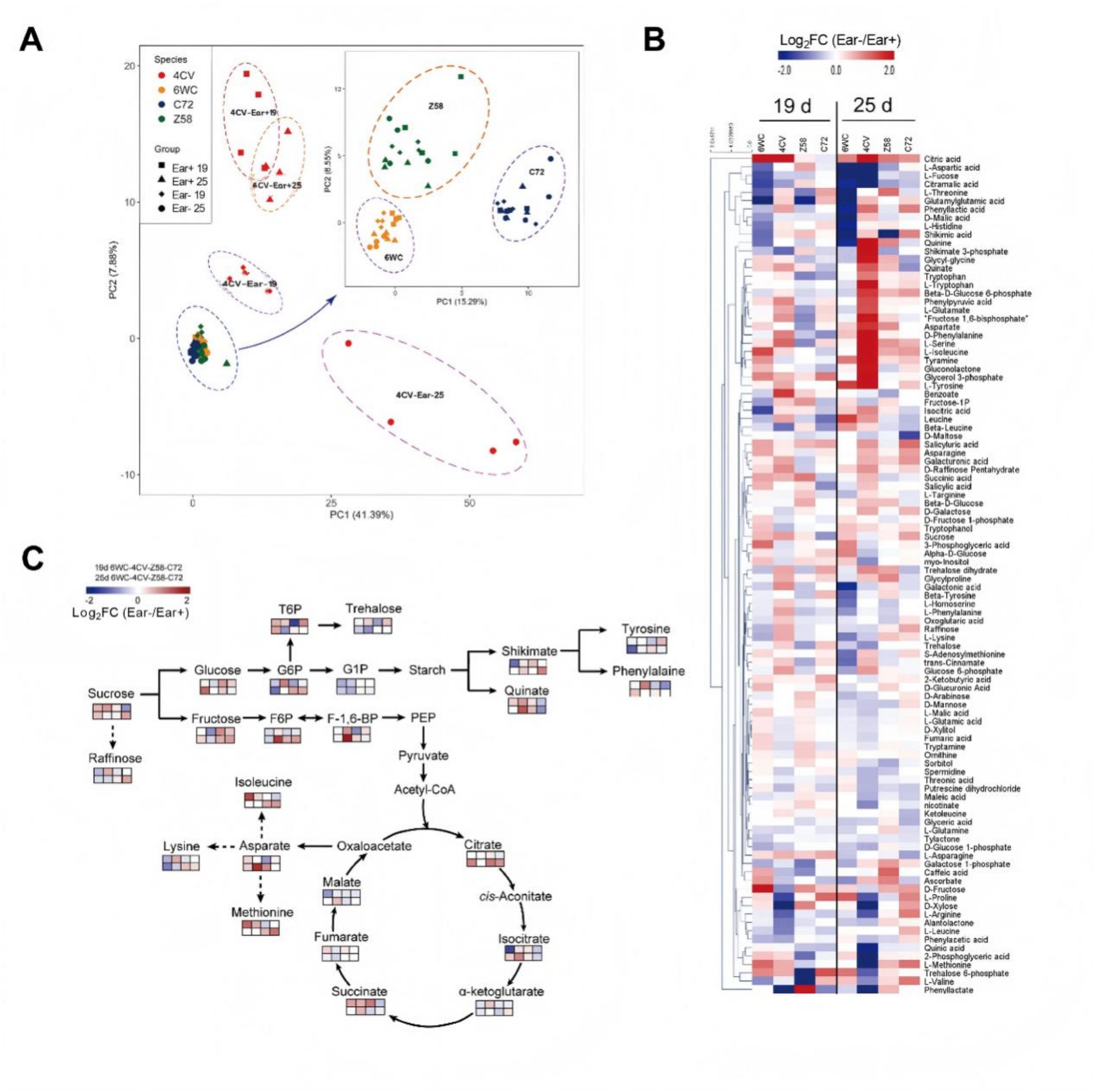

Supplementary Figure S6. Metabolite profiling of four maize inbred lines (PH6WC (6WC), PH4CV (4CV), Z58 (Z58) and C7-2 (C72)) under Ear+ and Ear- treatments.

(A) Principal Component Analysis (PCA). (B) Heatmap showing Log_2 fold change (Ear-/Ear+) of metabolites at 19 DAT (left) and 25 DAT (right). (C) Changes in metabolites involved in sugar metabolism and amino acids biosynthesis across genotypes and time points. Data are based on n = 4 biological replicates. DAT, days after treatment.

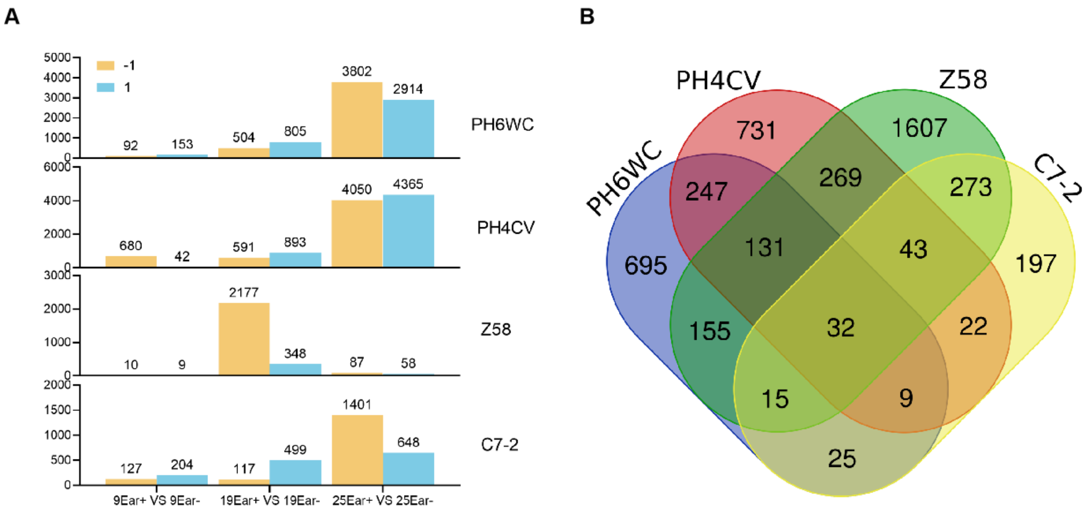

Supplementary Figure S7. Transcriptome profiling of four maize inbred lines under Ear+ and Ear- treatments.

(A) Number of differentially expressed genes (DEGs) at 9, 19, and 25 DAT. (B) Venn diagram showing DEG overlap across genotypes at 19 DAT. Data are based on $n = 3$ biological replicates. DAT, days after treatment. DEG, differential expression genes.

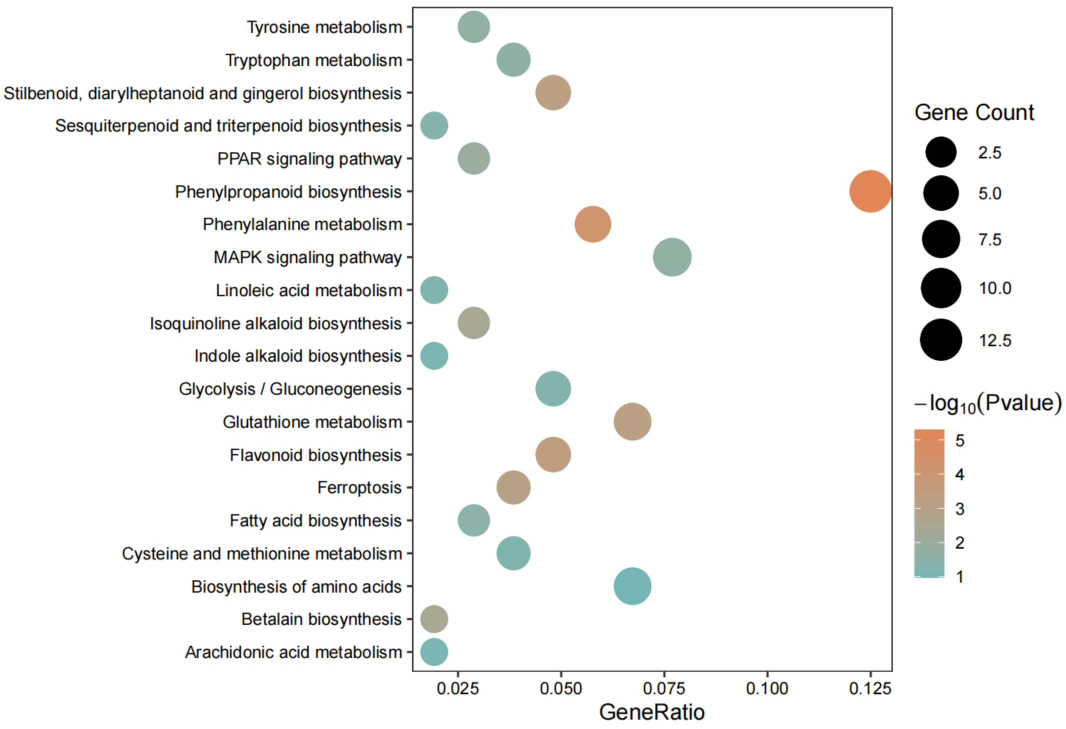

Supplementary Figure S8. KEGG pathway enrichment analysis of genes in Cluster 1.

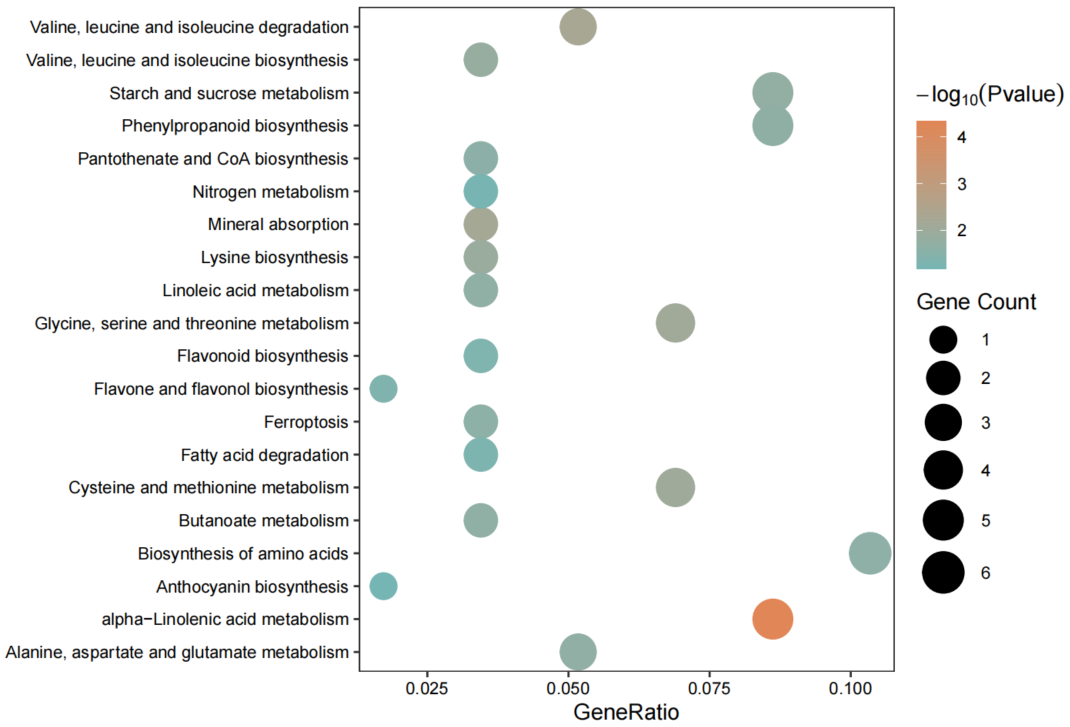

Supplementary Figure S9. KEGG pathway enrichment analysis of genes in Cluster 2.

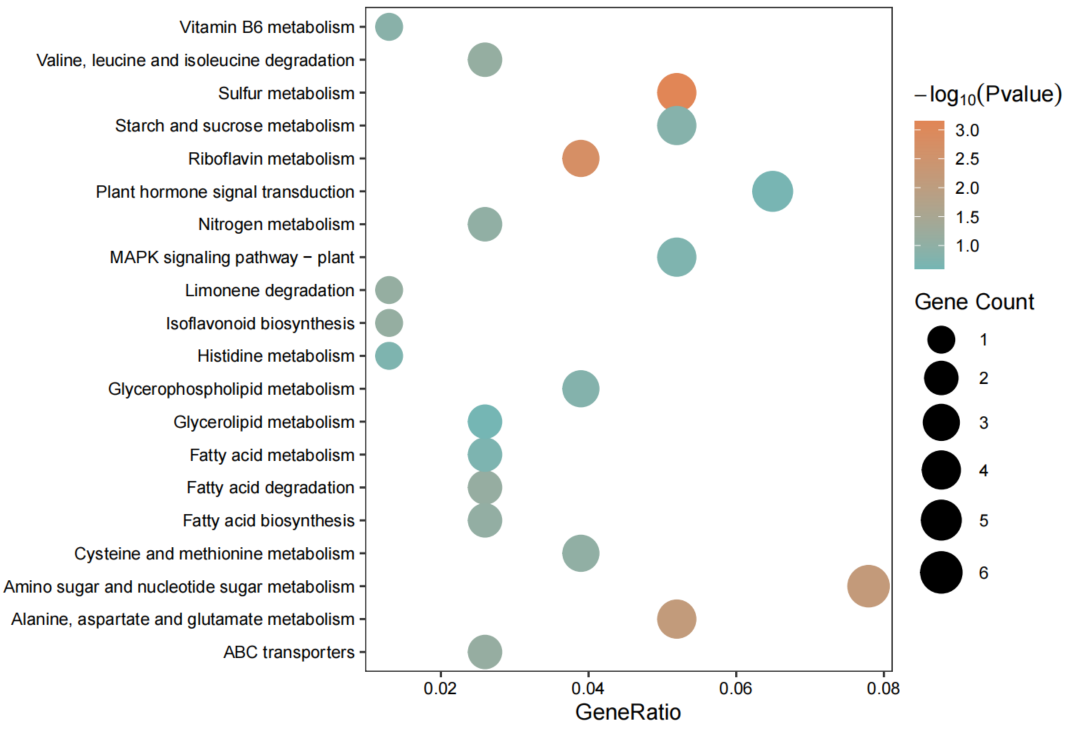

Supplementary Figure S10. KEGG pathway enrichment analysis of genes in Cluster 3.

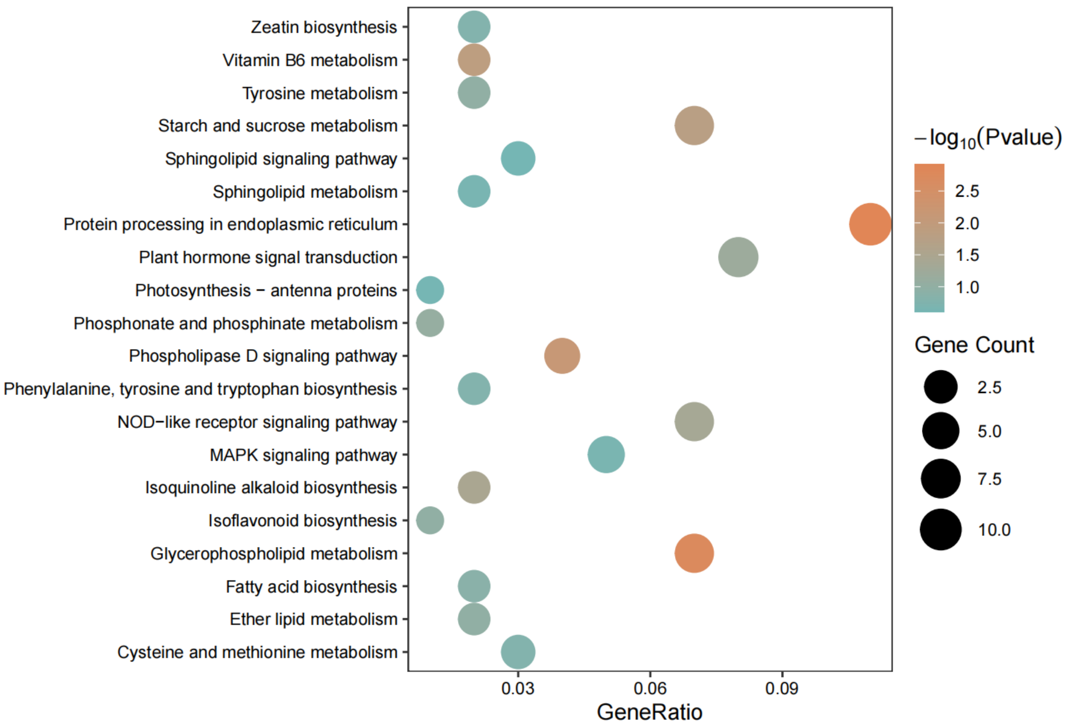

Supplementary Figure S11. KEGG pathway enrichment analysis of genes in Cluster 4.

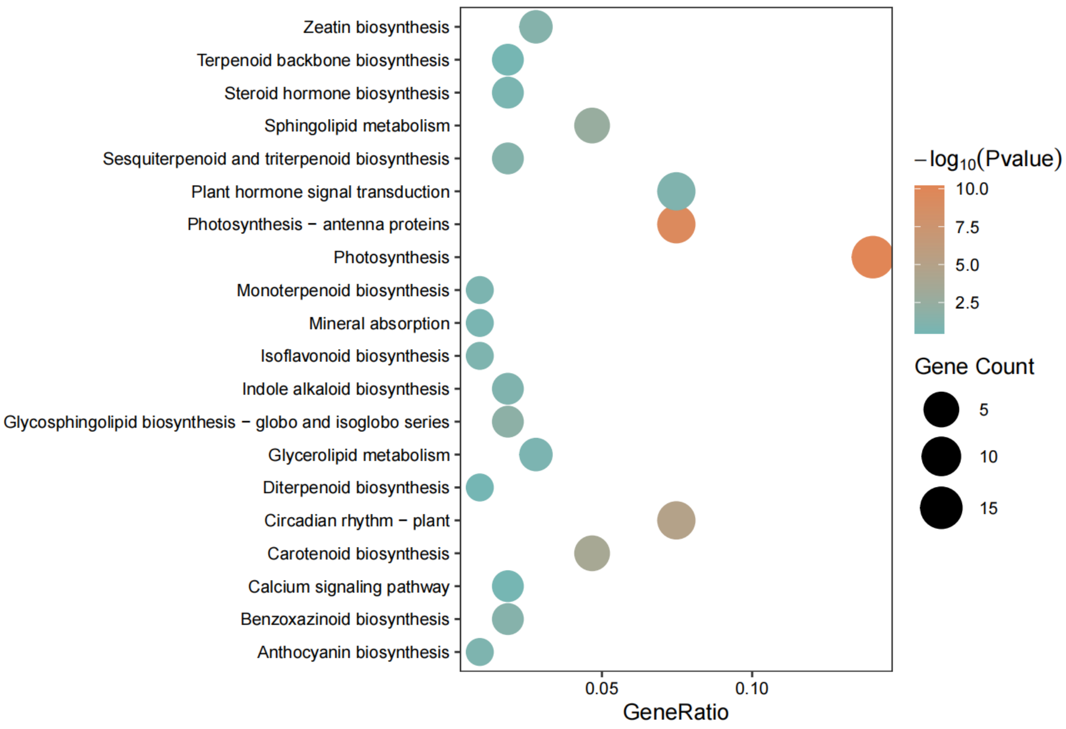

Supplementary Figure S12. KEGG pathway enrichment analysis of genes in Cluster 5.

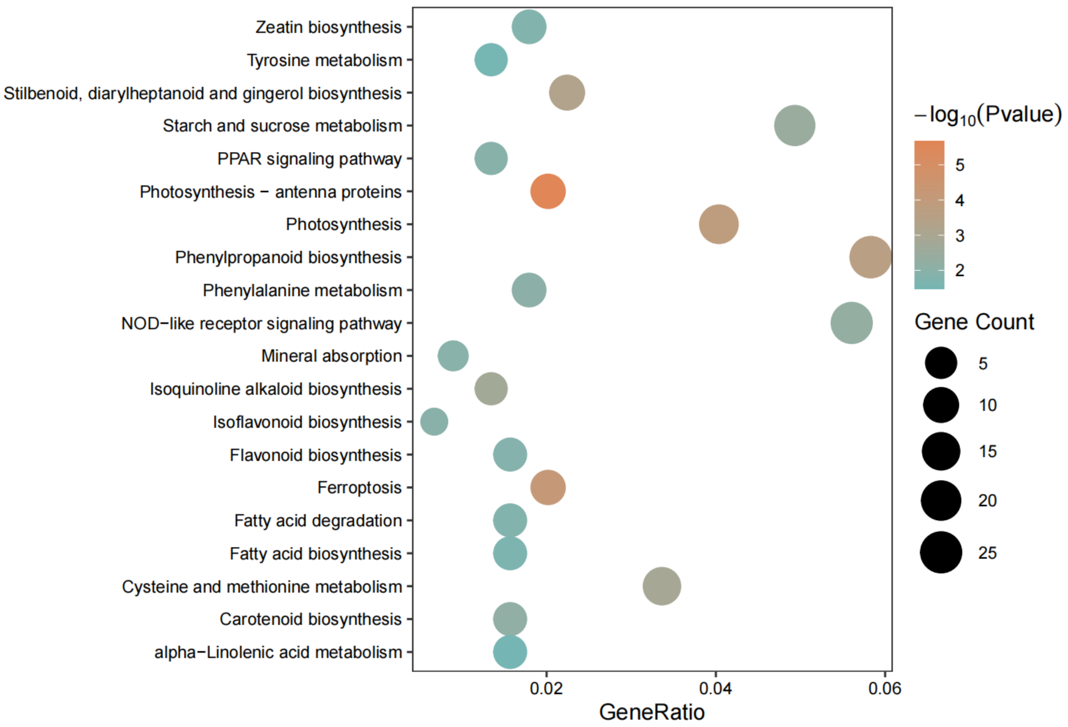

Supplementary Figure S13. KEGG pathway enrichment analysis of all 1,673 senescence-associated genes.

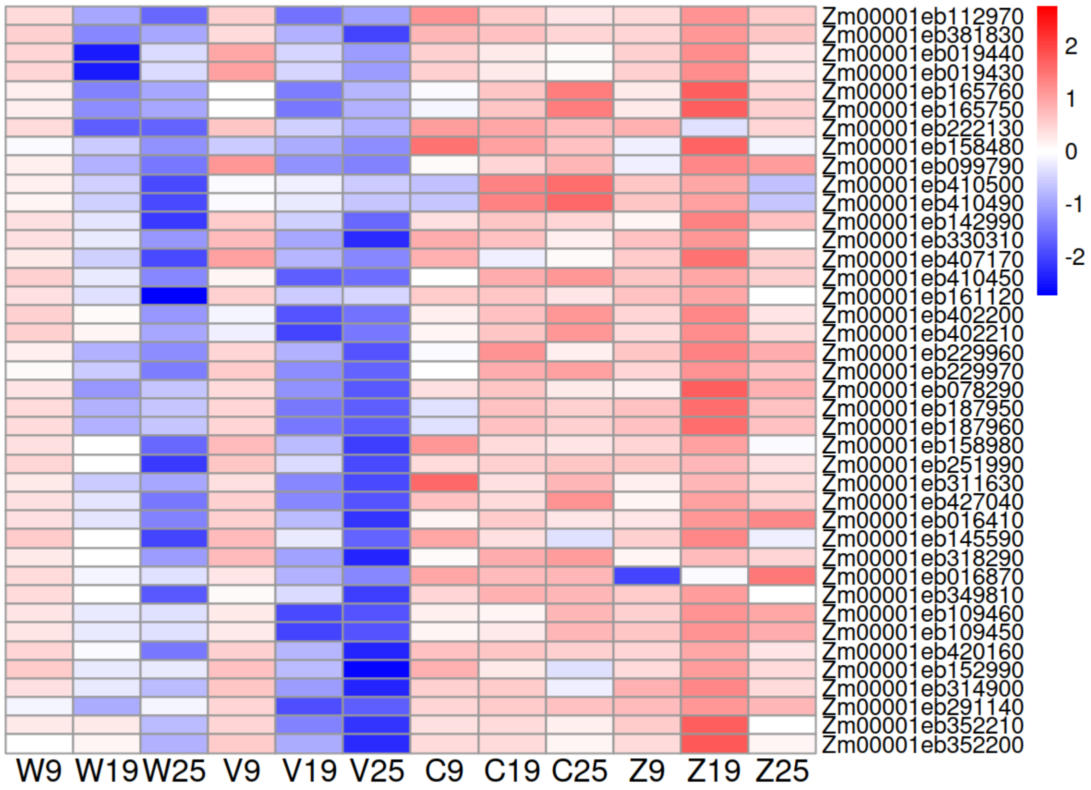

Supplementary Figure S14. Expression of key genes during Ear- induced senescence in maize.

Heatmap showing Log_2 FC in gene expression ratio of Ear-/Ear+ at 9, 19, and 25 DAT. Red indicates upregulated genes under Ear-; blue indicates downregulated genes. Gene IDs are listed on the right. DAT, days after treatment.

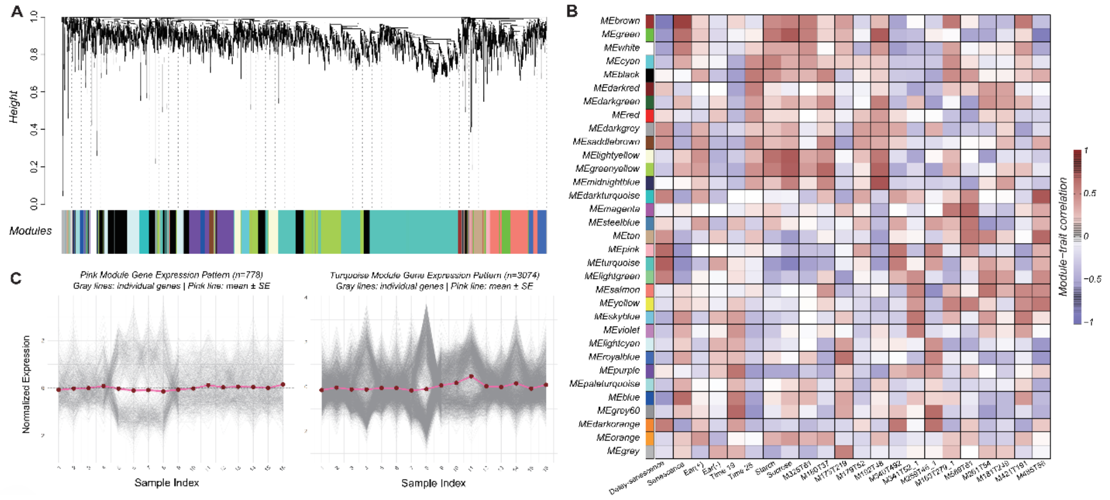

Supplementary Figure S15. Identification of gene co-expression modules and their correlation with traits.

(A) Gene clustering and module construction. (B) Correlation between traits and modules. Red indicates positive correlations, and purple indicates negative correlations. (C) Expression pattern of genes in the pink and turquoise modules.

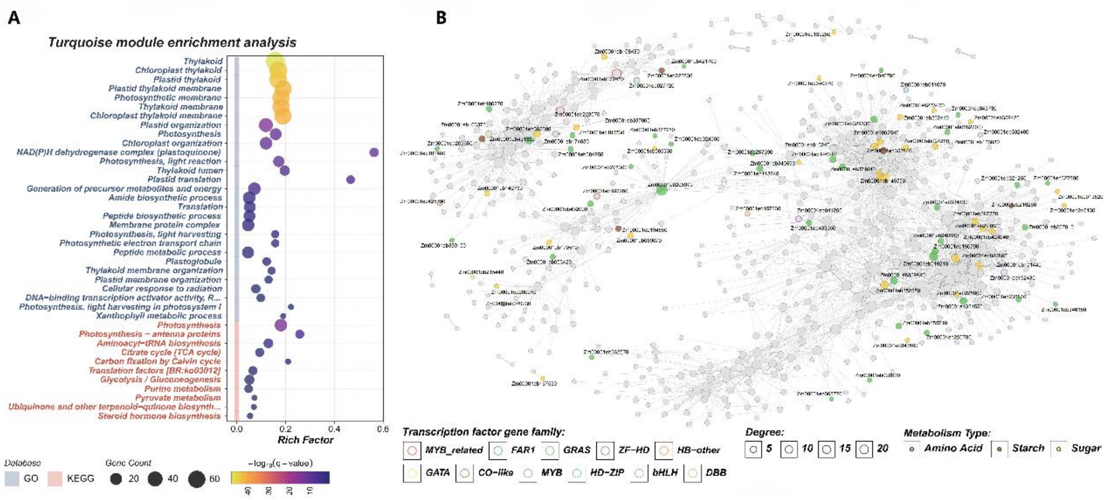

Supplementary Figure S16. Enrichment analysis and co-expression network of genes in the turquoise module.

(A) Enrichment analysis. (B) Co-expression network of genes in the turquoise module. Node size reflects connectivity, with larger nodes representing higher number of connections.

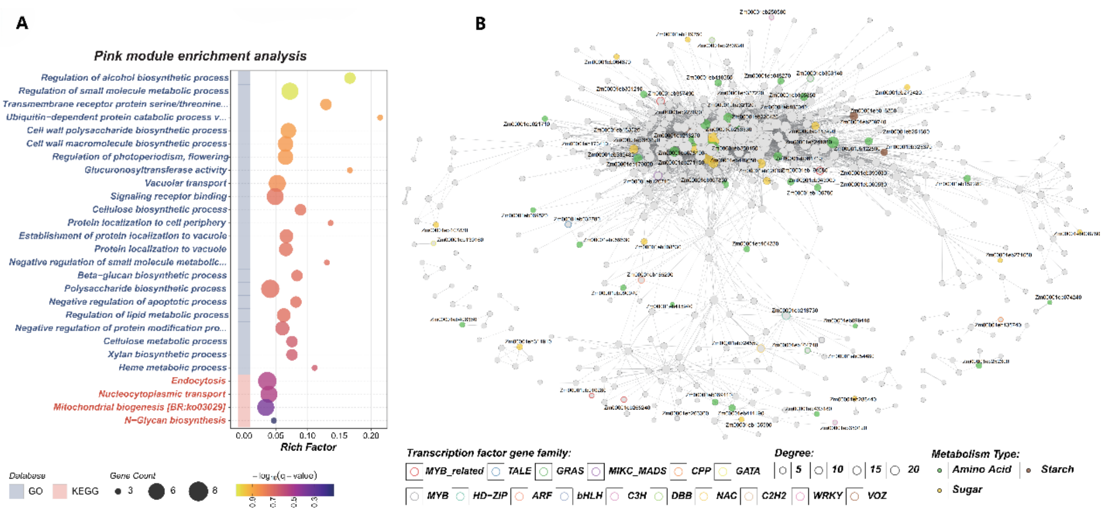

Supplementary Figure S17. Enrichment analysis and co-expression network of genes in the pink module.

(A) Enrichment analysis. (B) Co-expression network of genes in the pink module. Node size reflects connectivity, with larger nodes representing higher number of connections.

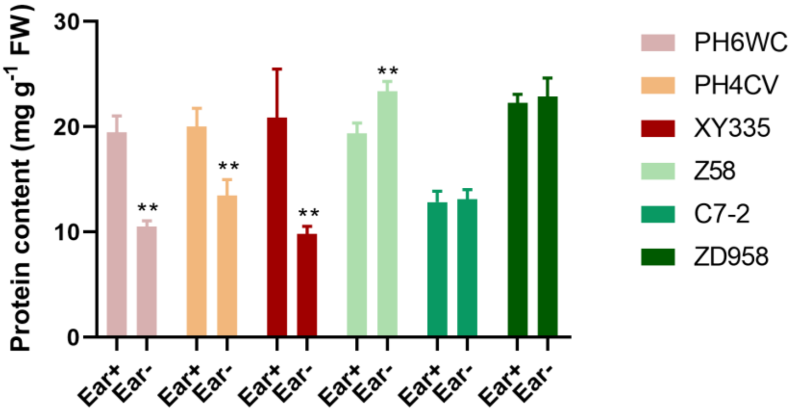

Supplementary Figure S18. Total leaf protein content in six maize genotypes at 25 DAT under Ear+ and Ear- treatments.

Data are presented as means ± SEM (*n* = 6). ** indicate statistically significant differences at t $P \leq 0.05$ and $P \leq 0.01$, compared with Ear+ (Student's *t*-test). DAT, days after treatment.
